# High-throughput microscopy exposes a pharmacological window in which dual leucine zipper kinase inhibition preserves neuronal network connectivity

**DOI:** 10.1186/s40478-019-0741-3

**Published:** 2019-06-04

**Authors:** Marlies Verschuuren, Peter Verstraelen, Gerardo García-Díaz Barriga, Ines Cilissen, Emma Coninx, Mieke Verslegers, Peter H. Larsen, Rony Nuydens, Winnok H. De Vos

**Affiliations:** 1 Laboratory of Cell Biology & Histology, Department of Veterinary Sciences, University of Antwerp, Antwerp, Belgium; 2 Department of Biology, Animal Physiology and Neurobiology, KU Leuven, Antwerp, Belgium; 3 Radiobiology Unit, Institute of Environment, Health and Safety, Belgian Nuclear Research Centre, Mol, Belgium; 4 Janssen Research & Development, a Division of Janssen Pharmaceutica NV, Beerse, Belgium

**Keywords:** Neuronal connectivity, Neuronal network, Synapse, Calcium imaging, High-content screening, Neurodegeneration, Antioxidant depletion, hTau.P301L

## Abstract

Therapeutic developments for neurodegenerative disorders are redirecting their focus to the mechanisms that contribute to neuronal connectivity and the loss thereof. Using a high-throughput microscopy pipeline that integrates morphological and functional measurements, we found that inhibition of dual leucine zipper kinase (DLK) increased neuronal connectivity in primary cortical cultures. This neuroprotective effect was not only observed in basal conditions but also in cultures depleted from antioxidants and in cultures in which microtubule stability was genetically perturbed. Based on the morphofunctional connectivity signature, we further showed that the effects were limited to a specific dose and time range. Thus, our results illustrate that profiling microscopy images with deep coverage enables sensitive interrogation of neuronal connectivity and allows exposing a pharmacological window for targeted treatments. In doing so, we revealed a broad-spectrum neuroprotective effect of DLK inhibition, which may have relevance to pathological conditions that ar.e associated with compromised neuronal connectivity.

**Electronic supplementary material:**

The online version of this article (10.1186/s40478-019-0741-3) contains supplementary material, which is available to authorized users.

## Introduction

Wiring the central nervous system demands precise formation and maintenance of neuronal connections. Functional connections between neurons are established by means of micron-sized interfaces, called synapses [19, 44]. Synaptic activity and the adjoined opening of gated calcium channels generate calcium transients that drive morphological changes such as dendritic growth and arborization, but which can also influence synapse strength [2]. This dynamic remodeling of both neurites and synapses fosters improved communication between neurons allowing synchronous functional activity, thereby reinforcing the overall connectivity of the neuronal network. All long-lasting adaptations of the brain, including learning, memory, addiction and chronic pain sensation, rely on the continuous remodeling of neuronal network connectivity [2]. And, disruption of this process is a hallmark of numerous neurological diseases, including schizophrenia, major depressive disorder and Alzheimer’s disease (AD) [40]. For example, the cognitive impairments witnessed in AD patients correlate with synapse and dendritic loss, as well as with a reduction of the brain activity, indicating an overall decrease in neuronal connectivity [16, 35, 60]. Therefore, therapeutic developments for neurodegenerative disorders are directing their focus to identifying regulators that promote neuronal connectivity or prevent the loss thereof.

The dense, three-dimensional organization and spatial heterogeneity of the brain makes studying neuronal connectivity in vivo a daunting task, which is not amenable to upscaling. Therefore, systematic screening efforts most often rely on simplified models such as neuronal cell cultures. Although some immortalized tumor cells (e.g.*,* SH-SY5Y human neuroblastoma cells, NT2 human teratocarcinoma cells, PC12 rat pheochromocytoma cells) can be differentiated to take on a neuron-like phenotype (as evidenced by e.g.*,* neurite outgrowth, expression of synaptic markers, induced or subtle spontaneous calcium activity), none fully recapitulate the full feature set of a physiologically connected neuronal network [1, 11, 18, 23, 27, 29]. Neurons derived from human induced pluripotent stem cell have a much higher translational value in comparison with other cell lines and primary cultures prepared from rodents, but the differentiation process is very labor and time intensive [15, 25, 36]. That is why, as yet, primary neuronal cultures represent the model of choice for genetic and pharmacological high-throughput screens [3, 6, 8, 9, 28, 31, 43, 53, 55]. However, most of these screens tend to focus on one or two specific readouts such as neuron number [6, 31], neurite outgrowth [3, 6, 8, 31] or synapse density [53, 55]. This reductionist approach can be misleading since it neglects multifactorial effects. For instance, measured changes in synapse density can be a direct reflection of real alterations in the number of synapses [28, 43], but could also be influenced by changes in the neurite density [62]. Furthermore, solely gauging morphological correlates may mask potential changes in functional connectivity. Indeed, it has already been shown that spontaneous activity in primary cultures does not scale linearly with synaptic density [5], and that morphological aberrations of primary neuronal networks do not always result in functional impairments [62]. In order to accurately quantify the overall neuronal connectivity in primary cultures, information gained from several readouts should thus be combined. Hence, instead of using one or a few descriptors, here we comprehensively assess the major morphological and functional correlates of neuronal network connectivity, and we integrate them to accurately map changes between subsequent maturation stages in vitro. In a targeted assay, we identified dual leucine zipper kinase (DLK) inhibition as a positive modulator of neuronal connectivity in unperturbed cultures and as a neuroprotector in cultures grown under suboptimal or compromised conditions.

## Materials and methods

### Preparation of primary neuronal cultures and pharmacological treatments

**Fig. 1:**
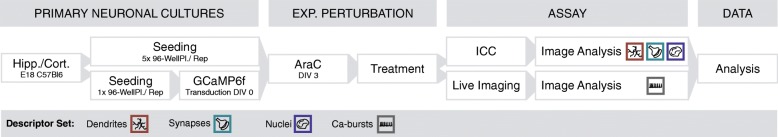
General workflow. Workflow of the microscopy-based pipeline. Hippocampi and/or cortices from WT E18 C57Bl6 mouse embryos were dissected, after which cell suspensions were created and seeded in 96-well plates. Cultures that would be used in the functional assay were transfected with an AAVDJ-hSyn1-GCaMP6f-nls-dTomato vector on 0 DIV. At 3 DIV, cultures were treated with arabinosylcytosine (AraC) to suppress the excessive growth of non-neuronal cells. Cultures were treated every three or four days. At fixed time points, cultures were either fixed and subjected to immunostaining, or used for live cell imaging to assess the morphological and functional characteristics, respectively

This study was carried out in accordance with the recommendations of the ethical committee for animal experimentation of the University of Antwerp (approved ethical file 2015–54). Hippocampi and cortex were dissected from WT E18 C57Bl6 mouse embryos in Hepes (7 mM)-buffered Hanks Balanced Salt Solution, followed by trypsin digestion (0.05%; 10 min; 37 °C) and mechanical dissociation by trituration through 2 pipette tips with decreasing diameter. After centrifugation (5 min at 200 g), the cell pellet was resuspended in Minimal Essential Medium supplemented with 10% heat-inactivated normal horse serum and 30 mM glucose. Cells were plated in Poly-D-Lysin-coated 96-well plates (Greiner Cell coat, μClear), at 30,000 cells/cm^2^, and kept in a humidified CO_2_ incubator (37 °C; 5% CO_2_). After 4 h, the medium was replaced with B27 (2%) supplemented Neurobasal medium, containing Sodium Pyruvate (1 mM), Glutamax (2 mM), glucose (30 mM) and PenStrep (0.5%). For anti-oxidant deprivation, the commercially available B27 supplement minus antioxidants was used. To suppress proliferation of non-neuronal cells, 1 μM arabinosylcytosine (AraC) was added in 25 μl Neurobasal-B27 medium at the third day after plating. Cell culture supplies were purchased from ThermoFisher. The following compounds were obtained from the in house Janssen pharmacy unless stated otherwise: rapamycin (Santa Cruz sc-3504), memantine, MK801, SAHA, tubastatin, GNE-8505, GNE-3511. These compounds were added in 25 μl Neurobasal-B27 medium at final concentrations of 0.01/0.1/1/10 μM. Compound treatment was initiated at 0 DIV (compound assay and DLK prevention experiments), 14 DIV (DLK rescue experiments) or 21 DIV (DLK aged cultures experiment), and repeated every three or four days. Control cultures received the same volume DMSO, which was always below 0.1% (Fig. 1).

### Immunocytochemistry

Cultures were fixed (2% PFA, 20 min, RT) at indicated time points (DIV) and immunocytochemically labeled for a dendrite marker (Chicken Polyclonal against MAP2, Synaptic Systems 188,006, 0.5 μg/ml), a presynaptic marker (Guinea Pig Polyclonal against Synaptophysin-1, synaptic systems 101,004, 0.5 μg/ml), a postsynaptic marker (Mouse Monoclonal against PSD-95, ThermoFisher MA1–046, 2 μg/ml) and a nuclear marker (DAPI, 5 μg/ml) (Fig. 1). In brief, the cultures were permeabilized with 1% Triton X-100 in blocking buffer (0.1% bovine serum albumin and 10% normal horse serum in PBS) for 10 min, followed by an overnight incubation with the primary antibodies at 4 °C in blocking buffer. After washing with PBS, secondary antibodies (Donkey-anti-Chicken-Cy5 / Donkey-anti-GuineaPig-Cy3 / Goat-anti-Mouse-AlexaFluorPlus488, 1 μg/ml) were added for 2 h at room temperature. Finally, DAPI was applied to the cultures for 10 min at a concentration of 2.5 μg/ml, followed by a PBS wash.

### AAV-mediated expression of GCaMP6f and MAPT-P301L

Functional connectivity was assessed by means of live cell calcium imaging of spontaneous neuronal activity (Fig. 1). At 0 DIV, a genetically encoded calcium indicator (GCaMP6f) along with a nuclear-localized red fluorescent protein (nls-dTomato) was introduced via AAV-mediated expression under the synapsin promoter (Addgene plasmid #51085 deposited by Jonathan Ting was packaged in-house with the AAV-DJ Helper Free Packaging System of Cell Biolabs; 0.01 μl crude lysate/well).

Overexpression of *hTau.P301L* was induced via AAV-mediated transduction of an expression vector under the synapsin promotor [7, 59] and were administered at 3 DIV at MOI 300.

### Western blot

Cultures were lysed with ice-cold RIPA buffer (Sigma R0278) supplemented with 5 mM ethylenediaminetetraacetic acid (EDTA) and complemented with phosphatase and protease inhibitors (HALT cocktail, Thermo Scientific #78445). Protein concentration was measured with the Bicinchoninic Acid Protein Assay kit (Sigma-Aldrich, BCA-1). Lysates were diluted in RIPA buffer to which Cy5 prelabelling was added (Amersham GE Healthcare 29,030,731). After 30 min on ice a sample mix was added consisting of a 5:2 ratio of NuPage LDS sample buffer (Life Technologies NP0008) and NuPAGE Sample reducing agent (Life Technologies NP00009). Samples were boiled (10 min, 70 °C) and 5 μg protein per sample was subjected to SDS-PAGE at 100-160 V (Criterion XT Bis-Tris gels, Bio-Rad 345–0124; MOPS SDS Running Buffer, Life Technologies, NP00001). Proteins were transferred to 0.2 μm nitrocellulose membranes for 10 min at 2.5A (Trans-Blot Transfer Pack, Bio-Rad 170–4159; Trans-Blot Turbo Blotting System, Bio-Rad), after which they were gently shaken for one hour in blocking buffer (2.5% non-fat dry milk (Santa Cruz ChemCruz sc-2325) in Tris Buffered Saline with 0.5% Tween 20). Next, the membranes were incubated overnight with the following primary antibodies: mouse anti-AT8 (in house JNJ, 1/2000), rabbit anti-c-Jun (Cell Signaling Technology #9165, 1/1000), rabbit anti-p-c-Jun (Ser 63) (Cell Signaling Technology #9261, 1/500), rabbit anti-p-c-Jun (Ser 73) (Cell Signaling Technology #3270, 1/500). After washing the blots with Tris Buffered Saline with 0.5% Tween 20, they were incubated with secondary antibodies for one hour: HRP conjugated donkey anti-rabbit (GE Healthcare Life Sciences NA934, 1/10000) and HRP conjugated sheep anti-mouse (GE Healthcare Life Sciences NA931,1/10000). After a final wash step, the total protein load was detected based on the Cy5 signal using the Amersham AI600 imager. Target proteins were detected by chemiluminescence with Supersignal West Femto Maximum Sensitivity Substrate (Thermo Scientific, 34,076). Images were acquired on the Amersham AI600 imager and analysed using the ImageQuant TL version 8.1 software (GE Healthcare).

### Image acquisition

Confocal images of immunostained cultures were acquired with an Opera Phenix High Content Screening System (PerkinElmer) using 40x water immersion lens (numerical aperture 1.1). At 488 nm excitation, the optical resolution of the system is 0.271 μm (and corresponding pixel size 0.149 μm), which is considerably larger than the distance of the synaptic cleft (15–25 nm), yet sufficiently small to allow signals of corresponding pre- and postsynaptic markers to partially overlap [64]. Per well, 15 fields were acquired in 4 channels (405, 488, 561 and 640 nm excitation) in 3-5 axial positions separated by a 1 μm spacing. Different fluorescence channels were separated using standard emission filters and dichroic mirrors.

Calcium imaging was performed on a spinning disk confocal microscope (20x, numerical aperture 0.75, UltraVIEW VoX, PerkinElmer) at 37 °C and 5% CO_2_. For each field, a 3-min recording (2 frames per second) of the calcium activity was acquired in the 488 nm channel, followed by a Z-stack of the nuclear NLS-dTomato signal in the 561 nm channel. Per well, 3 fields were imaged.

### Image processing and analysis

Image analysis was carried out in Acapella® (PerkinElmer) based on a template from Evotec, but a similar pipeline is available upon request for FIJI [12, 45]. The acquired images were read in per field of view. After maximum projection of the Z-stacks obtained from the MAP2 and DAPI channel, the nuclei were detected using a manually assigned threshold. Dendrites were identified using a rough (user-defined threshold) and fine (user-defined threshold after Frangi filtering [21]) segmentation. Neuronal nuclei were distinguished from non-neuronal based on a user-defined maximal projected area, minimal circularity and minimal occupancy in the dendrite mask. For both the dendrite network and nuclei a range of morphological and textural (object- and image-based) descriptors were extracted (Additional file 1: Table S1). Next, the dendrite mask and the neuronal nuclei mask were dilated and subtracted from each other to obtain a search region (i.e., dilated dendrites without neuronal nuclei) in which the pre- and postsynaptic spots were detected. The sharpest slice (based on the highest standard deviation of the intensity) from the presynaptic channel and corresponding postsynaptic channel were used for spot detection. The spots were first enhanced using a difference of Gaussian filter with a user-defined kernel size, after which a user-defined threshold was applied to segment the spots. To minimize noise contributions, only spots larger than 4 pixels were retained for further analysis. The resolution of the microscope setup does not allow determining the exact location of individual markers within a synapse, but this is not the intention of the assay. Instead, the lower resolution was exploited to define synapses as those objects that demonstrate an overlap of at least 1 pixel between the pre- and postsynaptic spots. We validated that the quantification of synapse density was not biased by chromatic aberration or the overlap criterion (Additional file 2: Figure S1). In addition to this object-based colocalization, the Pearson correlation of the pre- and postsynaptic channel was calculated as an intensity-based colocalization metric. Next to morphological and textural descriptors, the density of pre- and postsynaptic spots and synapses was calculated per measure of dendrite length or dentritic network area (Additional file 1: Table S1).

Calcium recordings were analyzed using a home-written MATLAB script, adapted from [10]. Briefly, individual neurons were segmented based on the NLS-dTomato signals, after which per object traces of the GCaMP6f fluorescence intensity over time were generated. After normalization, signal analysis returned parameters such as percentage of active neurons, frequency and amplitude of (synchronous) calcium bursts and burst correlation, which is the average of the Pearson’s correlation matrix between all neuron pairs in the field of view.

### Data integration and representation

Data analysis and representation was done in R Studio [13]. CSV files containing the field data were read and merged with the metadata according to the plate layout. Fields from the morphological assay lacking nuclei or dendrites were removed. False nuclear segmentations (e.g. debris) were identified and removed when the projected nuclear area exceeded the mean projected area by 5 standard deviations. After filtering of the data, well averages were calculated for the morphological data and were depicted as technical replicates. Data extracted from the functional assay was not averaged to not further reduce the number of data points and each recording was considered as a technical replicate. For both assays one biological replicate was considered as a primary culture derived from mouse embryos from one single WT C57Bl6 mother mouse. Z-scores of all descriptors were calculated using the mean and standard deviation within each experiment and replicate. PCA analyses were done on these z-scores using the *stats:*:*prcomp* function in R. Classification was done on the raw data using the *randomForest::randomForest* function in R. Both the control cultures of morphological and functional data were split in a training (2/3) and test (1/3) set. For each classifier the optimal number of trees and descriptors was determined using 10-fold cross validation, after which the optimal settings were used to classify the test set. The same training and test set was used to train and validate a linear discriminant analysis classifier (LDA, *MASS::lda* function in R).

The calculation of the connectivity score consisted of four steps; 1) first, the correlation of all descriptors with the culture age (DIV) was determined for all control conditions and averaged across six separate experiments; 2) next, the inter-correlation of the descriptors was calculated in a merged dataset including control conditions and treated cultures; 3) descriptors were then ranked according to their average correlation with culture age; 4) this ordered descriptor list, was then filtered top-down, by removing those descriptors with an absolute inter-correlation higher than 0.75 (Additional file 3: Figure S2). Channel descriptors that were sensitive to outliers (e.g.*,* intensity metrics) were removed as well. The final connectivity score was calculated as the average z-score of the subset of descriptors using their respective correlation with DIV as weights. When comparing control cultures with perturbed or treated cultures, z-scores were normalized to control or untreated suboptimal conditions within each experiment, replicate and time point.

All the data representations were built using the *ggplot2::ggplot* function in R. Mean and standard errors were visualized in bar and line plots. Indicated significance levels were based on pairwise Wilcoxon tests with Bonferroni correction, since normality and homoscedasticity of the data could not be assumed based on the Shapiro-Wilk and Bartlett test.

## Results

### Culture age correlates with both morphological and functional changes in primary neuronal networks

**Fig. 2:**
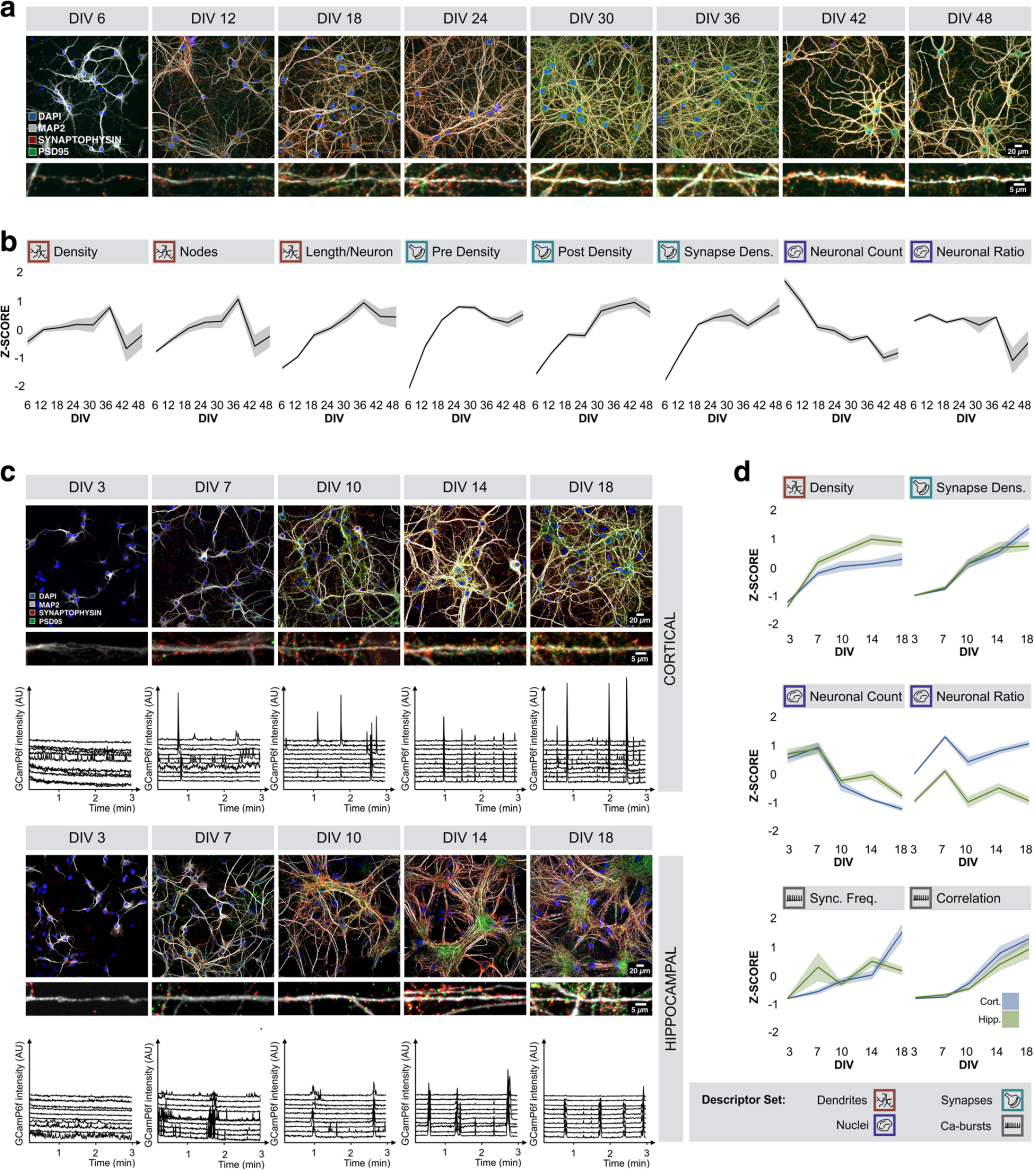
Culture age correlates with morpho functional changes. **a** Representative images of cortical cultures after fixation and immunocytochemistry at 6, 12, 18, 24, 30, 36, 42 and 48 DIV; **b** Dendrite network density and nodes increased gradually until 36 DIV, after which they decreased. The deterioration of the dendrite network was less pronounced when normalized to the number of neurons, which decreased over time. The presynaptic density and synapse density increased during the first 18 DIV. The density of postsynaptic spots increased gradually. The ratio between neuronal and non-neuronal nuclei remained more or less the same until 36 DIV (n_bio_ = 2 x n_tech_ = 6); **c** Representative immunocytochemistry images and Ca^2+^- traces (of 10 neurons) for a more resolved experiment (3, 7, 10, 14, 18 DIV) of primary hippocampal and cortical cultures; **d** Similar trends are observed in selected morphological descriptors for hippocampal and cortical cultures (n_bio_ = 3 x n_tech_ = 6). Synchronous activity increased from DIV 10 onwards (n_bio_ = 3 x n_tech_ = 6)

When cultured in vitro, primary neurons form dense dendritic networks and develop functional activity, epitomized by a synchronicity of intracellular calcium fluctuations [5, 63, 65]. To determine the temporal evolution of these changes, we cultured cortical neurons for 48 days in vitro (DIV) and quantified a variety of morphological parameters after fixation and immunostaining at 6-day intervals (Fig. 2a, Additional file 4: Figure S3). We thereby made a distinction between descriptors that inform on one of three major categories, namely the dendrite network, the synapse markers, or the nuclei (Additional file 1: Table S1). Up to 36 DIV, the neuronal network became denser, as exemplified by an increase in dendrite network density (the area of the field of view covered by dendrites) and the number of nodes (branch points) in the network (Fig. 2b). At later time points, the dendrite network deteriorated. The presynaptic spot density – inferred from synapthophysin immunolabeling – increased rapidly up to 18 DIV, after which it stabilized only to decrease after 30 DIV. The postsynaptic spot density – as gauged from PSD-95 immunolabeling – followed a more gradual evolution and reached a maximum at 42 DIV. In line with this, the relative number of overlapping pre- and postsynaptic spots, a proxy for synapse density, stabilized at 18 DIV (Fig. 2b). The number of neuronal nuclei (and thus neurons) gradually decreased with culture age. The ratio of neuronal cells versus non-neuronal (glial) cells remained relatively constant until 36 DIV, after which it significantly decreased, predominantly due to a stronger loss of neurons vs. glial cells (Fig. 2b). Thus, this multiparametric analysis showed that cortical neurons develop progressive morphological connectivity up to 36 DIV with the strongest evolution taking place between 3 and 18 DIV.

We next wondered whether this evolution was generic to cultures derived from different brain regions. Therefore, we compared cortical with hippocampal cultures and quantified changes with a higher time resolution (3, 7, 10, 14 and 18 DIV) (Fig. 2c). For both neuronal culture types, we found similar trends in the previously mentioned descriptors (e.g.*,* dendrite density, synapse density, nuclear count) (Fig. 2d), suggesting that they both become morphologically more connected during this culture period. However, there were also culture type-dependent differences. Hippocampal neurons displayed greater tendency to form tight dendrite bundles than cortical neurons. This clustering occurred especially at later time points (Fig. 2c and Additional file 5: Figure S4a). Hippocampal cultures also showed a stronger postsynaptic marker intensity (Additional file 5: Figure S4a). Arguably one of the most striking differences between both cultures, was a lower proportion of neurons in hippocampal cultures and a comparatively higher number of glial cells. Since glial cells display larger, flattened nuclei with less pronounced chromocenters, this was reflected by a higher average area and lower average (spot-like) texture of nuclei in hippocampal cultures (Additional file 5: Figure S4a,b).

Recognizing that morphological measures do not necessarily report on functional changes in connectivity [62], we complemented the morphological analysis with a previously optimized live cell calcium imaging assay [10]. A very similar evolution was found for both culture types, with virtually no synchronous bursting activity - expressed as the correlation between calcium bursts of all active neurons in the field of view - at 3 or 7 DIV, and progressively more synchronous activity at later time points (Fig. 2c,d). This was also reflected – albeit to a lesser extent – in other functional descriptors such as the synchronous bursting frequency (Fig. 2d). Thus, we conclude that primary hippocampal and cortical cultures form neuronal networks that become both morphologically and functionally more connected with culture age, at least up to 18 DIV.

### Neuronal culture states can be distinguished by their morphofunctional signature

**Fig. 3:**
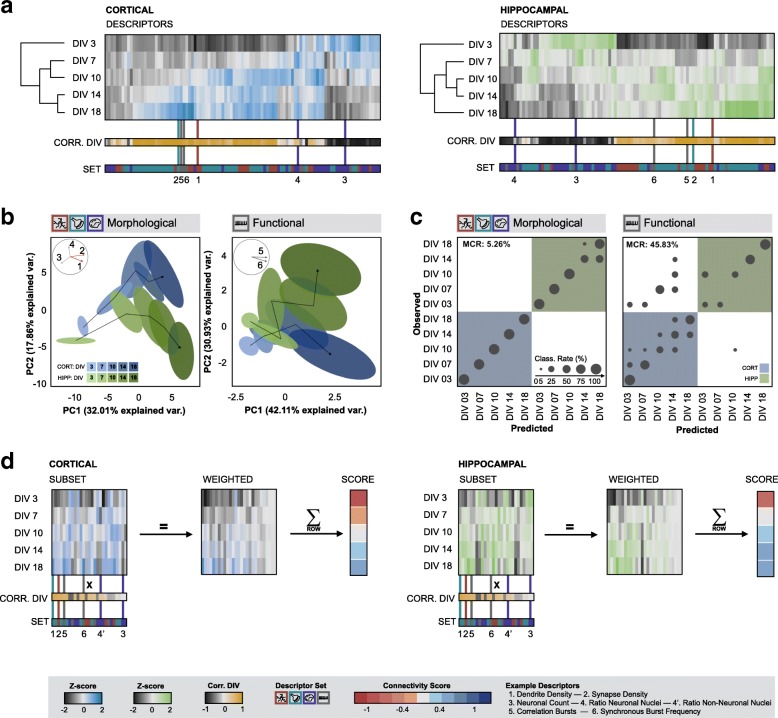
Cultures can be clustered and classified based on their morphofunctional signature. **a** Hierarchical clustering based on z-scores of all descriptors. The correlation of the descriptors with culture age (DIV) is indicated (black/orange color coded) as well as the descriptor set to which they belong; **b** PCA based on morphological descriptors distinguished hippocampal from cortical cultures. In a biplot of the first two principal components, data points cluster according to culture time and follow distinct temporal trajectories along the direction of decreasing nuclear count and increasing dendrite- and synapse density (n_bio_ = 3 x n_tech_ = 6). A biplot of PCA on functional descriptors could only differentiate early (3–10 DIV) from later time points (14–18 DIV) (n_bio_ = 3 x n_tech_ = 6) (Ellipses show the 67% confidence interval); **c** Confusion matrices and classification results using a random forest classifier. The misclassification rate (MCR) of the classification based on morphological descriptors was 5% (n_bio_ = 3 x n_tech_ = 6) and much lower than the MCR of the classification based on functional data (45%) (n_bio_ = 3 x n_tech_ = 6); **d** The z-scores of a subset of descriptors were multiplied with their respective correlation with culture age (weighted z-scores) and summed to obtain a connectivity score that allows facile interpretation of the degree of connectivity at a given time point (n_bio_ = 3 x n_tech_ = 6)

Having established that both hippocampal and cortical neurons showed distinct, yet consistent morphofunctional changes with culture age, we asked whether we could retrieve this evolution in an unsupervised manner from the extracted descriptors. Hierarchical clustering on the entire data set revealed that each condition disposed of a distinct descriptor profile, with many descriptors showing a high correlation with culture age (47% of the descriptors had an absolute correlation with DIV > 0.5) (Fig. 3a). To navigate more easily through the high-dimensional descriptor space, we applied principal component analysis (PCA). Despite significant variability between individual biological replicates (Additional file 6: Figure S5a), the pooled data showed a clear and reproducible pattern on the combined morphological descriptor set. When plotting the two first principal components, each cell type displayed a distinct trajectory of instances clustered according to culture age (DIV) (Fig. 3b). We also tested PCA on the separate morphological descriptor classes (nuclei, dendrite, synapse), but no single class could unequivocally separate both cell type and culture time as well, indicating that a combined descriptor set yielded the most powerful discriminatory fingerprint (Additional file 6: Figure S5b). PCA of the functional descriptor set did not lead to well-defined trajectories either, most likely due to the fact that synchronous activity only surfaced from 10 DIV onwards (Fig. 3b).

Given the clear separation in PC space, we next asked whether we could use the information, contained within the descriptors, to predict culture age per cell type. To this end, we trained a random forest classifier (RFC) using different parameter settings and selected the best performing classifier using 10-fold cross-validation on a training set. Then, we determined the misclassification rate (MCR) on a test set (Fig. 3c). While individual descriptor sets yielded classifiers with MCRs above 14% (Additional file 6: Figure S5c), a classifier based on all morphological descriptors returned a MCR of 5%. Comparable results were obtained using a trained linear discriminant analysis-based classifier (Additional file 6: Figure S5d). Not unexpectedly, the performance of a classifier that was solely based on functional descriptors was poor (MCR > 45%). Yet, the fact that a morphological descriptor set could be used to untangle and even predict culture age, suggests that an integrated signature could be a measure for the degree of neuronal connectivity.

Due to a different experimental setup and number of technical replicates, morphological and functional data could only be combined at the level of the biological replicate. This reduced the number of data points drastically and precluded reliable PCA or RFC. However, we reasoned that the exclusion of functional descriptors from a connectivity analysis could lead to a bias under specific challenged conditions. Indeed, when we treated cortical cultures with the N-methyl-d-aspartate sensitive glutamate receptor (NMDA-R) antagonist MK801, we found an adverse impact on functional connectivity that was significant (*p* = 9.1E-6) at 18 DIV, without showing significant changes in key morphological descriptors at that time point (dendrite density: *p* = 1, synapse density: p = 1, neuronal count: p = 1) (Additional file 7: Figure S6). Similarly, treatment of hippocampal cultures with arabinosylcytosine (AraC), which selectively blocks astrocyte proliferation, evoked a significant, adverse effect on functional connectivity (burst correlation) at 14 and 18 DIV (14 DIV: *p* = 2.7E-4, 18 DIV: *p* = 2.5E-2), a significant positive effect on dendrite outgrowth at 18 DIV (14 DIV: p = 1, 18 DIV: *p* = 4.1E-4) and no significant effect on synapse density at 14 and 18 DIV (14 DIV: p = 1, 18 DIV: p = 1) (Additional file 8: Figure S7). The same treatment also caused a significant shift in the non-neuronal nuclei count with culture age (3 DIV: *p* = 9.9E-9, 7 DIV: *p* = 1.4E-5, 10 DIV: *p* = 1.0E-4, 14 DIV: *p* = 4.0E-8, 18 DIV: p = 1.4E-5), suggesting that nuclear descriptors carry relevant information on the culture state as well (Additional file 8: Figure S7). Therefore, we sought an approach to integrate all morphofunctional descriptor classes in such a way that it intuitively reports on connectivity changes, but without disregarding putative off-target effects (Fig. 3d). To avoid redundancy, we excluded descriptors that showed high inter-correlation (> 0.75), whilst giving priority to descriptors that correlated best with culture age (Additional file 3: Figure S2). From this subset, a weighted average was calculated using the correlation with culture age as weights. This resulted in a single metric which we refer to as *connectivity score* (Fig. 3d). The connectivity score is sensitive to changes in any of the four descriptor classes (as demonstrated on the MK801 or AraC data, Additional file 9: Figure S8), allowing it to instantly report on deviations from the basal (unperturbed) connectivity trajectory.

### Focused assay identifies DLK as a positive modulator of neuronal network connectivity

**Fig. 4:**
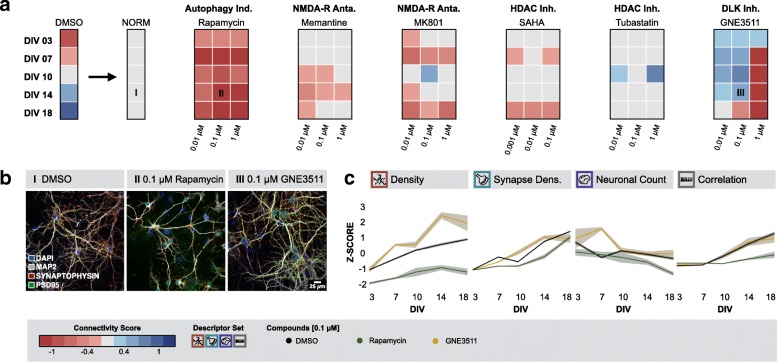
Focused compound assay identifies DLK as positive modulator of neuronal connectivity. **a** Connectivity score map after normalization to culture age-matched controls. Rapamycin is a strong negative modulator of neuronal connectivity and GNE3511 exerts a lasting positive effect on neuronal connectivity. (Morph.: n_bio_ = 3 x n_tech_ = 5 - Func.: n_bio_ = 3 x n_tech_ = 6, except for GNE3511: Morph.: n_bio_ = 2 x n_tech_ = 6 - Func.: n_bio_ = 2 x n_tech_ = 9); **b** Example images of control cultures and cultures treated with 0.1 μM rapamycin or GNE3511 (14 DIV) (I, II and III in panel a); **c** Z-scores showing the effect of 0.1 μM rapamycin or GNE3511 on dendrite density, synapse density, neuronal count (rapamycin: n_bio_ = 3 x n_tech_ = 5, GNE3511: n_bio_ = 2 x n_tech_ = 6) and correlation of calcium bursts (rapamycin: n_bio_ = 3 x n_tech_ = 6, GNE3511: n_bio_ = 2 x n_tech_ = 9)

Using the connectivity score as primary readout, we subsequently initiated a focused assay to expose small molecule regulators of neuronal network connectivity. Given the higher culture yield, better reproducibility, and lower tendency to cluster, we chose to continue with cortical cultures. A rational selection of putative molecular targets (mTOR, NMDA-R, histone deacetylases (HDAC), DLK) was made based on their published involvement in neurodegenerative conditions and for each target, at least one compound (rapamycin, memantine, MK801, suberoylanilide hydroxamic acid (SAHA), tubastatin, GNE3511) was selected [24, 38, 41, 46, 51, 56, 72]. Concentrations were based on previously reported IC_50_ values from neuronal cell-based assays [46, 74–77], within a dose range of 3 log scales. These compounds were tested in unperturbed cultures to expose the dose range that does not exert negative effects in basal conditions. Rapamycin, inhibitor of mTOR, was used as negative control, based on the notion that mTOR activity is crucial in the developmental stages of the neurite network as well as for the synaptic strength at later stages [34]. In total 792.000 images (49.500 fields, 4 channels and 5 z-slices) were acquired for this experiment. To reduce variability and aid legibility, scores of challenged conditions were normalized to their culture age-matched controls (Fig. 4). As anticipated, rapamycin had an overt negative impact on neuronal connectivity across the tested dose range. Conversely, the DLK inhibitor GNE3511 had an unequivocal positive effect at different culture ages except DIV 18 at doses below 1 μM. Higher concentrations induced neurotoxicity, which could be expected according to previously reported IC_50_ values [46]. These findings were also confirmed – albeit to a weaker extent - when using a RFC based on morphological descriptors of the control cultures to predict the degree of connectivity (Additional file 10: Figure S9). Thus, the connectivity score revealed a pharmacological dose and time window in which DLK inhibition promoted connectivity in otherwise unperturbed cultures.

### DLK inhibition has both neuro-protective and -restorative potential

**Fig. 5:**
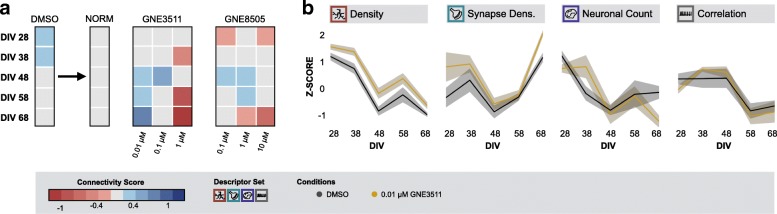
DLK inhibition can partially prevent the age-related loss of connectivity in culture. **a** Connectivity score map showing that chronic treatment with 0.01 μM GNE3511 could prevent the age-related loss of connectivity. Other effects were not lasting or neurotoxic (Morph.: n_bio_ = 1 x n_tech_ = 6 - Func.: n_bio_ = 1 x n_tech_ = 9); **b** Z-scores of example descriptors (Morph.: n_bio_ = 1 x n_tech_ = 6 - Func.: n_bio_ = 1 x n_tech_ = 9)

The observation that DLK inhibition exerted a positive effect on neuronal connectivity in primary cultures under basal conditions, drove us to test whether the same treatment was able to prevent or slow down the gradual connectivity loss observed in old cultures (Fig. 5 and Additional file 11: Figure S10). To this end, we incubated cortical cultures with two different DLK inhibitors (GNE3511 and GNE8505) every 3 days from DIV 21 onwards and followed them up to 68 DIV. A positive effect on the connectivity score was found in older cultures (> = DIV48) treated with a low dose (0.01 μM) of GNE3511 [46]. Yet, this effect was not consistently recapitulated with GNE8505 [47].

**Fig. 6:**
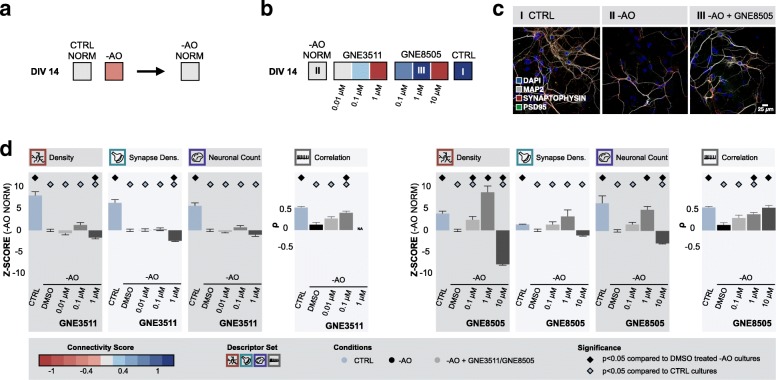
DLK inhibition has neuroprotective potential in cultures deprived from antioxidants. **a** Primary cultures deprived from antioxidants (-AO), display impaired neuronal network connectivity at DIV 14 (Morph.: n_bio_ = 2 x n_tech_ = 6 - Func.: n_bio_ = 2 x n_tech_ = 9); **b** Normalized connectivity scores of cultures deprived of antioxidants show that treatment from 0 DIV onwards with DLK inhibitors (GNE3511 and GNE8505) could prevent connectivity loss at 14 DIV (excl. Highest concentration) (Morph.: n_bio_ = 2 x n_tech_ = 6 - Func.: n_bio_ = 2 x n_tech_ = 9); **c** Representative images of control cultures, -AO cultures and -AO cultures treated with 1 μM GNE8505 (I, II and III in panel b); **d** Bar plots showing z-scores normalized to -AO cultures of dendrite density, synapse density and neuronal count (n_bio_ = 2 x n_tech_ = 6), as well as the absolute number of correlated calcium bursts (n_bio_ = 2 x n_tech_ = 9). Significant differences compared to control cultures or cultures deprived from antioxidants are indicated (*p* < 0.05, pairwise Wilcoxon test with Bonferroni correction)

Next, we tested whether DLK inhibition could also prevent cultures from degenerating when grown under sub-optimal or challenged conditions. Taking advantage of the fact that neurons do not dispose of well-established antioxidant protection mechanisms and are therefore highly susceptible to oxidative stress [17, 61], we grew cortical cultures in medium without antioxidants (-AO). This had an adverse impact on neuronal connectivity, as reflected by a decreased connectivity score and increased misclassification by RFC from 7 DIV onwards (Additional file 12: Figure S11ab). To reduce the experimental load, we limited our analyses to one time point where the effect of antioxidant depletion was sufficiently clear, namely at 14 DIV (Fig. 6a). Chronic treatment with 0.1 μM GNE3511 could slightly improve the connectivity score in comparison to the DMSO-treated cultures deprived from antioxidants (Fig. 6b), but the increase in the z-score of key morphological descriptors with respect to DMSO-treated -AO cultures was not significant (dendrite density: *p* = 6.8E-1, synapse density: *p* = 1, neuronal count: p = 1)(Fig. 6d). Conversely, chronic treatment with DLK inhibitor GNE8505 (0.1 μM and 1 μM) drastically improved the connectivity score in comparison with DMSO-treated -AO cultures (Fig. 6b,c). The z-scores of dendrite density, synapse density and neuronal count after treatment with 1 μM GNE8505 increased such that they no longer differed significantly from or were higher than those measured in unperturbed control cultures (dendrite density: *p* = 7.9E-1, synapse density: *p* = 1.6E-2, neuronal count: *p* = 4.9E-1) (Fig. 6d). These results suggest a neuroprotective effect of GNE8505 in this sub-optimal growth condition. To validate the underlying molecular mechanism, we quantified cJun (a transcription factor in the c-Jun N-terminal kinase (JNK) mediated stress response pathway) levels with western blot. In accordance with the described *modus operandi* of DLK inhibition [54, 66], GNE8505 lowered both phosphorylated (ser73) and non-phosphorylated cJun levels (Additional file 13: Figure S12a).

**Fig. 7:**
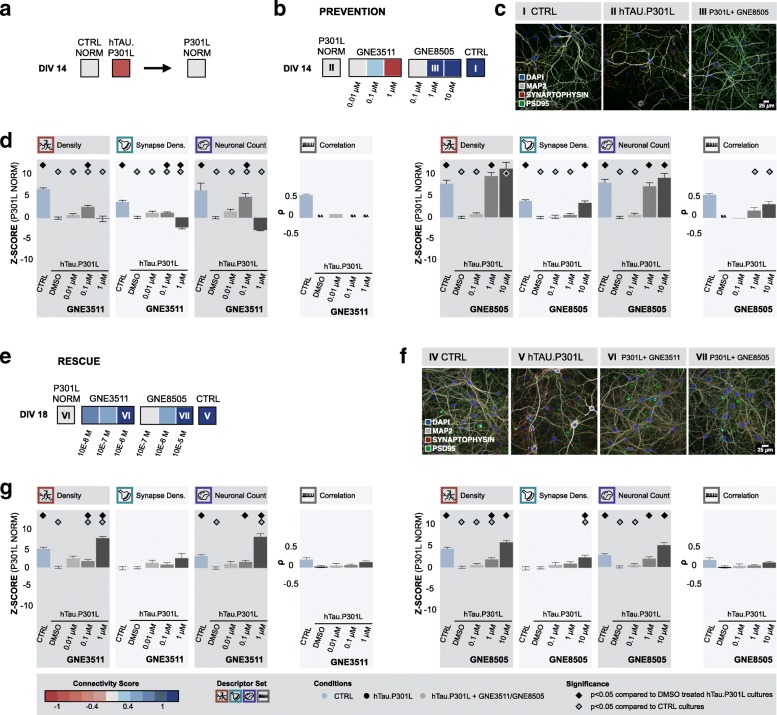
DLK inhibition has both neuro-protective and -restorative potential in cultures overexpressing hTau.P301L. **a** Cultures overexpressing hTau.P301L, showed impaired neuronal network connectivity (Morph.: n_bio_ = 2 x n_tech_ = 6 - Func.: n_bio_ = 2 x n_tech_ = 9); **b** Continuous treatment with DLK inhibitors prevented loss of neuronal connectivity of the hTau.P301L model according to the normalized connectivity scores (Morph.: n_bio_ = 2 x n_tech_ = 6 - Func.: n_bio_ = 2 x n_tech_ = 9); **c** Representative images of control cultures, cultures overexpressing hTau.P301L and hTau.P301L cultures treated with 1 μM GNE8505 (I, II and III in panel b); **d** Bar graphs showing z-scores of morphological descriptors (n_bio_ = 2 x n_tech_ = 6) as well as the absolute number of correlated calcium bursts (n_bio_ = 2 x n_tech_ = 9). Significant differences compared to control cultures or cultures overexpressing hTau.P301L are indicated (p < 0.05, pairwise Wilcoxon test with Bonferroni correction); **e** DLK inhibition rescues cultures overexpressing hTau.P301L after connectivity loss had already started (Morph.: n_bio_ = 1 x n_tech_ = 6 - Func.: n_bio_ = 1 x n_tech_ = 6); **f** Representative images of control cultures, cultures overexpressing hTau.P301L and hTau.P301L cultures treated with 1 μM GN3511 or 10 μM GNE8505 (IV, V, VI and VII in panel e); **g** Bar graphs showing z-scores of morphological descriptors (n_bio_ = 1 x n_tech_ = 6) as well as the absolute number of correlated calcium bursts (n_bio_ = 1 x n_tech_ = 6). Significant differences compared to control cultures or cultures overexpressing hTau.P301L are indicated (p < 0.05, pairwise Wilcoxon test with Bonferroni correction)

To assess the specificity of the neuroprotective action of DLK inhibition, we next introduced a completely different type of challenge to the cultures. We selectively altered microtubule stability through overexpression of hTau.P301L (at 3 DIV). Upon overexpression, a progressive decline in neuronal connectivity was witnessed from 10 DIV onwards (Additional file 12: Figure S11c,d). This correlated with the accumulation of hyperphosphorylated tau, as revealed by AT8 immunoblotting (Additional file 13: Figure S12b) and an upregulation of non-phosphorylated and phosphorylated (ser63) cJun levels (Additional file 13: Figure S12a). When supplementing challenged hTau.P301L cultures with GNE8505, we found a positive impact on the connectivity score in comparison to DMSO treated hTau.P301L cultures at DIV 14 (Fig. 7a,b). After treatment with 1 μM or 10 μM GNE8505, z-scores of dendrite density, synapse density (10 μM only), and neuronal count showed no significant differences with untreated control cultures (dendrite density: 1 μM p = 1 and 10 μM p = 1, synapse density: 1 μM *p* = 4.4E-6 and 10 μM p = 1, neuronal count: 1 μM p = 1 and 10 μM p = 1) (Fig. 7c,d), suggesting protection from the genetic insult. In line with -AO cultures, western blot revealed a significant decrease on both phosphorylated and non-phosphorylated c-Jun levels after GNE8505 treatment (Additional file 13: Figure S12a).

To test whether DLK inhibition could not only prevent but also reverse this process, we repeated the experiments in cultures overexpressing hTau.P301L, but this time we only started treating cultures with DLK inhibitors at a time point where connectivity loss was already manifesting (DIV14). Strikingly, in cortical cultures that overexpressed hTau.P301L, a rescue of connectivity loss could be observed at DIV 18 (Fig. 7e). After treatment of hTau.P301L-overexpressing cells with 10 μM GNE8505, dendrite density reached the same level as for untreated control cultures (*p* = 1.9E-1) (Fig. 7f,g). Treatment with 1 μM GNE3511 even significantly increased the density area above control levels (*p* = 1.4E-3) (Fig. 7f,g). Thus, we conclude that DLK inhibition is able to protect cortical cultures from diverse challenges and even partially rescue cultures from ensuing connectivity loss.

## Discussion

The continuous remodeling of the neuronal network is crucial for learning, memory and behavior, but is disrupted in several psychiatric and neurodegenerative disorders [40, 49]. Identification of novel therapeutic targets requires a method that is able to quantify neuronal network connectivity over time with high accuracy and throughput. To our knowledge, the high-throughput analysis proposed in this paper is the first to comprehensively gauge neuronal connectivity in primary cultures by including descriptor sets reporting on the dendrite network, synapse markers, nuclei and calcium bursting activity. Other high-throughput studies aimed at finding regulators of neuronal connectivity [3, 6, 9, 28, 43], have mainly focused at one or few readouts such as neuron number [6] and neurite outgrowth [3, 6], synapse density [53, 55] or calcium responses [65]. The strength of an integrative approach lies in the fact that it can account for a number of potential sources of bias. For instance, when only considering synapse density, observed changes could be the result of true variations in the synapse number in cultures with comparable dendrite  density [28, 43], but they could equally arise from an altered dendrite density with preserved synapse count [62], an increased density of only one or both synaptic partners (pre/post) [43], an increased spot size of one or both synaptic partners, or an altered clustering of neuronal somata and fasciculation of dendrite bundles. Furthermore, the inclusion of calcium data as a proxy for spontaneous electrical network activity allows determining whether alterations in the network morphology also have a functional impact and vice versa. For example, previously published data by our group shows that overexpression of human tau in hippocampal cultures decreased the neurite density but increased the synaptophysin density [62]. At a functional level, the percentage of active neurons and frequency of synchronous bursts decreased while the synchronicity was preserved. This underscores the argument that conclusions may vary depending on the descriptors used and that an integrative approach is desirable.

In line with other studies, we found an increase in dendrite density and synapse density with culture age, and - although not linear – a strong increase in the synchronous bursting behavior of the network at later time points (> 10 DIV). The time scales used in literature range from DIV6 to DIV21, which corresponds with the time range we identified in our initial experiment to be the time span in which dendrite density, synapse density and functional activity increased drastically. The decrease in neuronal cell count with culture age confirmed previous findings [28, 33], but we now also showed that certain treatments affect cell culture composition, in particular the ratio of nuclear and glial cells (e.g.*,* AraC). To our knowledge, no other connectivity studies have made such a distinction and it shows the value of including nuclear descriptors.

When applying PCA to the unfiltered morphological descriptor set, we unveiled distinct temporal trajectories [14], suggesting that the culture age could serve as an indicator of connectivity. This was confirmed by the fact that a RFC could predict culture age with an accuracy of 95%. To include functional parameters in the analysis, we defined a comprehensive connectivity score. Using this score as readout, we confirmed that inhibition of mTOR activity (using rapamycin) impairs the development of the dendritic network [34, 58]. Nevertheless, rapamycin has been used to slow down or block neurodegeneration in mouse models of Alzheimer’s, Parkinson’s and Huntington’s disease, through the induction of autophagy that cleared accumulated autophagosomes and/or aggregated proteins [4, 37, 52]. Therefore, an extension of this work could focus on assessing the effect of rapamycin on neuronal connectivity under conditions of induced toxic protein aggregation. Antagonists of NMDA-R, such as memantine and MK801, were also tested. These antagonists have the potential to block the excessive NMDA-R activity in many neurodegenerative diseases, hereby reducing the increased calcium influx in the neurons [41, 51]. NMDA-R antagonists can however also block the normal function of these receptors, which was suggested by the overall negative effect on the connectivity score for MK801 (in which the functional descriptors had a major contribution). The score indicated that memantine treatment had only a slight negative impact on neuronal connectivity in comparison with MK801, which may be the result of memantine having a shorter dwell time on the NMDA-R then MK801 [41]. Tubastatin, a HDAC inhibitor that improved cognitive deficits in mouse models of Alzheimer's Disease by improving microtubule stability [72], did not have a negative, nor a neurotrophic effect on the overall neuronal connectivity in control conditions, and may therefore be further explored in compromised conditions. Treatment with another HDAC inhibitor, SAHA, resulted in a decreased neuronal connectivity at later time points. This may be due to the fact that SAHA targets multiple HDAC classes, whereas tubastatin only targets HDAC6 [69].

The sole compound that showed a clear neurotrophic effect in unperturbed primary cultures was an inhibitor of DLK, which is why we chose to pursue the efficiency of this compound in compromised conditions. DLK, also known as MAP3K12, is an upstream regulator of the JNK stress response pathway, which becomes activated in both acute and chronic neurodegenerative conditions [54]. Its activation induces a broad transcriptional injury response (via c-Jun and ATF4) [66]. The fact that it is upstream in this highly conserved pathway makes that DLK inhibition has a broad action range, at least in preclinical models. Pharmacological or genetic DLK inhibition protects against excitotoxicity [50], growth factor deprivation [22, 42, 68], amyloid and tau pathology [32, 38], nerve crush and traumatic brain injury [66, 71] retinal ganglion cell degeneration [67, 68] and SOD1-mediated neurodegeneration [38]. At this moment, one inhibitor is in Phase I clinical trial for Amyotrophic Lateral Sclerosis (ALS) by Genentech (Roche) [30]. Our results now show that also in unperturbed primary cortical cultures, DLK inhibition results in enhanced morphofunctional connectivity. Yet, it should be noted that the inhibitor treatment started 4 h after plating. It is possible that the cells already experienced stress during the dissociation procedure, which could have been attenuated by DLK inhibition.

We found that DLK inhibition could prevent and even rescue neurodegeneration induced by hTau.P301L overexpression. This aligns with literature data where double mutant mice (Tau^P301L^;DLK^cKO^) show attenuated cell loss in the subiculum compared to single mutant (Tau^P301L^) mice without having altered tau pathology [38]. Our in vitro approach now also shows that hTau-P301L-induced neuron loss is accompanied by impaired dendrite formation, synapse density and functional activity and that this can be prevented and rescued by pharmacological DLK inhibition. DLK inhibition reduced the increased levels of unphosphorylated and phosphorylated (ser63) c-Jun that were detected in cultures overexpressing hTau.P301L.

The omission of anti-oxidants from the culture medium of primary cortical neurons resulted in gradual impairment of morphofunctional connectivity, which could be prevented by DLK inhibition as well. Neurons are particularly sensitive to reactive oxygen species (ROS) and most neurodegenerative disorders are associated with increased oxidative stress [61], making our in vitro model attractive for drug screening. In line with our results, it was described before that the SOD1^G93A^ mouse model for ALS exhibits neuronal cell death due to an impaired oxidative stress defense and that this is accompanied by aberrant JNK pathway activation [38]. Double mutant mice (SOD1^G93A^;DLK^cKO^) show enhanced neuronal survival and myelinization as well as reduced neuroinflammation compared to single mutants (SOD1^G93A^), resulting in enhanced grip strength and longer life span of the former model. In AO-depleted cell culture, we did not find evidence for the activation of the JNK pathway, but we did detect a downregulation of the pathway upon DLK inhibition.

Finally, we also verified whether DLK inhibition could sustain morphofunctional connectivity in aging cultures, as it is known that JNK signaling is elevated in the aging brain [70, 73]. However, the effect was rather limited. Increased variability across aged cultures and reduced sensitivity of the morphological readout due to the very high density of dendrites covering most of the culture plate could have masked a potentially crisper effect of DLK inhibition on ageing. Across experiments, we found differential efficacy of both DLK inhibitors in various growth conditions. These discrepancies are likely the result of different treatment duration and starting times, as well as different target selectivity of both inhibitors [46, 54]. Literature reports on the selectivity of both compounds show that GNE-3511 had a K_i_(DLK) below 0.5 nM and a pJNK IC_50_ of 30 nM, which was considerably lower than the IC_50_ for other kinases (MKK4 > 5.000, MKK7 > 5.000, JNK1 = 129, JNK2 = 514, JNK3 = 364, MLK2 = 767, MLK3 = 602 nM), except for MLK1 (IC_50_ = 67.8) [46]. For GNE-8505, the K_i_(DLK) and pJNK IC_50_ values were higher than for GNE-3511 (4 nM and 144 nM, respectively), yet the selectivity for DLK over other kinases was markedly better (MKK4 > 5.000, MKK7 > 5.000, JNK1 > 10.000, JNK2 > 10.000, JNK3 > 10.000, MLK1 = 3.500, MLK2 = 5.150, MLK3 > 10.000 nM). This illustrates that the DLK inhibitors used in this study can target other kinases, especially in the higher dose range, which might explain the differential effects of both compounds.

To increase the sensitivity of the current approach, it could be advantageous to include more synapse markers so as to better map the full landscape of synapse types (e.g.*,* inhibitory vs excitatory). To bypass spectral limitations, one could resort to the use of narrow-emission band labels, such as quantum dots [20], or divert to cyclic immunofluorescence staining protocols [26, 39]. The latter would however lower the throughput and put a higher demand on downstream analyses (e.g.*,* image registration). A particular caveat of the current approach was the high variability of the functional readout. To strengthen the sensitivity of this assay one could resort to imaging larger cell populations by increasing the field of view. Previous studies have revealed the co-existence of separate networks of high connectivity within a large neuronal network that may not always fire in sync [48, 57] Identification of stratified connectivity patterns may therefore expose more subtle modifications of the functional connectivity. Selective addition of chemical stimuli (e.g.*,* glutamate) could further unveil cell type specificity as well as differences in spontaneous and induced functional activity [10].

## Conclusions

In conclusion, we have shown that morphofunctional profiling of primary cultures using deep coverage microscopy allows accurate quantification of neuronal connectivity in vitro. We established a connectivity score, including morphological and functional correlates, to identify modulators of neuronal connectivity. With our approach we were able to expose a dose and time window for DLK inhibitors that evoked positive effects on neuronal connectivity and could even rescue challenged cultures. Therefore, the current approach holds promise for identifying pathways and treatments that preserve or rescue neuronal connectivity in neurodegenerative disorders.

## Additional files

Additional file 1: **Table S1.** All descriptors for the dendrite network, the synapse markers, the nuclei and functional (calcium) activity. Measurements are reported per field of view. (PDF 10993 kb)

Additional file 2: **Figure S1.** Opera Phenix system chromatic aberration and overlap criteria of pre- and postsynaptic markers. **(a)** Fluorescent beads of 0.1 and 0.5 μm diameter were recorded with a 40x water immersion objective (NA = 1.1) at an image resolution of 0.149 μm/pixel. Dashed white lines show the axis at which the intensities of the 488 nm (typically used for PSD95) and 561 nm (typically used for Synaptophysin) excitation channel were measured. Quantifications in XY and XZ show that the shift between both channels lies below 1 pixel for both bead types. (*n* = 25 beads); **(b)** An interpolated shift of 0.6 pixel (~ shift calculated for 0.1 μm beads in panel (a)) or a one-pixel shift of the PSD95 channel to correct for the chromatic aberration does not alter the synapse density calculation in images of cortical cultures, fixed at 3/7/10/14/18 DIV (n_tech_ = 6); **(c)** Different criteria for synapse detection (1, 2 or 3 pixels overlap between pre- and postsynaptic spots) alter the absolute synapse numbers slightly, but do not affect the relative change across DIVs (n_tech_ = 6). (PDF 10993 kb)

Additional file 3: **Figure S2.** Descriptor selection for the connectivity score. A subset of descriptors was selected based on the correlation with the DIV and inter-correlation between descriptors. First, the descriptors were ranked according to their correlation with the culture age. This rank was used to subsequently add descriptors to the final subset, making sure that the inter-correlation within descriptors of the subset did not exceed 0.75. Intensity descriptors were excluded from the descriptor set, because they were too sensitive to outliers. (PDF 10993 kb)

Additional file 4: **Figure S3.** Culture age correlates with morphological changes. Individual channels and composite of representative images of cortical cultures after fixation and immunocytochemistry at 6, 12, 18, 24, 30, 36, 42 and 48 DIV. (PDF 10993 kb)

Additional file 5: **Figure S4.** Differences between hippocampal and cortical cultures. **(a)** Dendrite width and postsynaptic intensity are higher in hippocampal cultures. The ratio of neuronal nuclei is higher in cortical cultures compared to hippocampal cultures that have more non-neuronal nuclei. This is reflected in the average texture and area of the nuclei, since neuronal nuclei are smaller and have a more spot-like phenotype (stronger texture) than non-neuronal nuclei (n_bio_ = 3 x n_tech_ = 6); **(b)** Representative images of both neuronal (red) and non-neuronal (yellow) nuclei in cortical and hippocampal cultures. (PDF 10993 kb)

Additional file 6: **Figure S5.** Despite inter-replicate variability, cultures can be clustered and accurately classified. **(a)** Representative images of both hippocampal and cortical cultures at different DIV for 3 different replicates; **(b)** PCA based on different descriptor sets for individual replicates. PCA based on the integrated descriptor set resulted in the best clustering (n_bio_ = 3 x n_tech_ = 6) (Ellipses show the 67% confidence interval); **(c)** Confusion matrices of classification results using a random forest. The misclassification rate (MCR) was lowest using the integrated morphological descriptor set (n_bio_ = 3 x n_tech_ = 6); **(d)** Confusion matrices of classification results using a linear discriminant analysis. The MCR, compared to RFC, was lower for morphological data, but higher for functional data (n_bio_ = 3 x n_tech_ = 6). (PDF 10993 kb)

Additional file 7: **Figure S6.** Functional descriptors entail unique information about neuronal connectivity. MK801 treatment (yellow) impaired the functional activity significantly at 18 DIV, that was not reflected in the morphological data (Morph.: n_bio_ = 3 x n_tech_ = 5 - Func.: n_bio_ = 3 x n_tech_ = 6). Significant differences between control and treated cultures are indicated (*p* < 0.05, pairwise Wilcoxon test with Bonferroni correction). (PDF 10993 kb)

Additional file 8: **Figure S7.** Nuclear descriptors entail unique information. AraC treatment (yellow) had a major negative impact on nuclear descriptors during the whole time range, while other descriptors showed only transient effects (e.g.*,* dendrite density) or negative effects on later time points (e.g.*,* correlation of the calcium bursts) (Morph.: n_bio_ = 3 x n_tech_ = 6 - Func.: n_bio_ = 3 x n_tech_ = 6). Significant differences between control and treated cultures are indicated (p < 0.05, pairwise Wilcoxon test with Bonferroni correction). (PDF 10993 kb)

Additional file 9: **Figure S8.** Connectivity scores are sensitive to changes in dendrite, synapse, nuclear and functional descriptors. **(a)** Connectivity scores of MK801-treated cultures showed greater differences with DMSO-treated cultures at later time points when based on the integrated dataset when compared to the scores only based on morphological data. (Morph.: n_bio_ = 3 x n_tech_ = 5 - Func.: n_bio_ = 3 x n_tech_ = 6); **(b)** Connectivity scores of AraC treated cultures revealed greater connectivity impairments in comparison with DMSO treated cultures when including nuclear descriptors (Morph.: n_bio_ = 3 x n_tech_ = 6 - Func.: n_bio_ = 3 x n_tech_ = 6). (PDF 10993 kb)

Additional file 10: **Figure S9.** Classification of morphological data confirms findings based on connectivity score. A RFC that was trained on pooled DMSO treated cultures revealed a negative impact of rapamycin on neuronal connectivity as can be seen from the cultures that were misclassified and were assigned a culture age that was lower than the actual culture age (red). Treatment with 0.01 μM and 0.1 μM of GNE3511 could however improve the neuronal connectivity (green) (n_bio_ = 3 x n_tech_ = 5 except for GNE3511: n_bio_ = 2 x n_tech_ = 6). (PDF 10993 kb)

Additional file 11: **Figure S10.** Extended culture age reduces neuronal connectivity**.** Connectivity scores based on z-scores from cortical cultures grown for an extended period of time. Neuronal connectivity increased during the first two weeks, after which it stagnated up to five and a half weeks. From DIV 38 onwards age-related loss of neuronal connectivity was detected (Morph.: n_bio_ = 1 x n_tech_ = 6 - Func.: n_bio_ = 1 x n_tech_ = 9). (PDF 10993 kb)

Additional file 12: **Figure S11.** Impaired neuronal connectivity in suboptimal conditions. **(a)** Connectivity scores indicated that antioxidant deprivation (-AO) in primary cultures had a negative impact on neuronal network connectivity form 7 DIV onwards (Morph.: n_bio_ = 2 x n_tech_ = 6 - Func.: n_bio_ = 2 x n_tech_ = 9); **(b)** A RFC that was trained on morphological data of pooled DMSO treated cultures confirmed the negative impact of antioxidant deprivation (red) (n_bio_ = 2 x n_tech_ = 6); **(c)** Cultures overexpressing hTau.P301L, showed a decreasing neuronal connectivity from 10 DIV (Morph.: n_bio_ = 2 x n_tech_ = 12 - Func.: n_bio_ = 2 x n_tech_ = 9); **(d)** Classification results based on morphological data confirmed the negative effect of hTau.P301L overexpression on neuronal connectivity (red) (n_bio_ = 2 x n_tech_ = 12). (PDF 10993 kb)

Additional file 13: **Figure S12.** Western blot analyses of (phosphorylated) Jun and AT8. **(a)** Western blot showed an increase in total c-Jun and phosphorylated c-Jun (Ser 63) in cultures overexpressing hTAU.P301L. Treatment with GNE8505 reduced c-Jun and phosphorylated c-Jun in control, antioxidant deprived (-AO) and hTau.P301L cultures (excl. Phosphorylated c-Jun Ser 63 in control and -AO cultures) (n_bio_ = 1 x n_tech_ = 1); **(b)** Western blot showed an increase in hyperphosphorylated (AT8) tau in cultures overexpressing hTau.P301L (n_bio_ = 1 x n_tech_ = 1). (PDF 10993 kb)

## Abbreviations

AraC: Arabinosylcytosine
DIV: Days in vitro
DLK: Dual leucine zipper kinase
EDTA: Ethylenediaminetetraacetic acid
HDAC: Histone deacetylase
JNK: c-Jun N-terminal kinase
LDA: Linear discriminant analysis
MCR: Misclassification rate
NMDA-R: N-methyl-d-aspartate sensitive glutamate receptor
PCA: Principal component analysis
RFC: Random forest classifier
SAHA: Suberoylanilide hydroxamic acid

## References

[1] Agholme L, Lindström T, Kågedal K, Marcusson J, Hallbeck M (2010). An in vitro model for neuroscience: differentiation of SH-SY5Y cells into cells with morphological and biochemical characteristics of mature neurons. J Alzheimers Dis.

[2] Bading H (2013). Nuclear calcium signalling in the regulation of brain function. Nat Rev Neurosci.

[3] Blackmore MG, Moore DL, Smith RP, Goldberg JL, Bixby JL, Lemmon VP (2010). High content screening of cortical neurons identifies novel regulators of axon growth. Mol Cell Neurosci.

[4] Bové J, Martínez-Vicente M, Vila M (2011). Fighting neurodegeneration with rapamycin: mechanistic insights. Nat Rev Neurosci.

[5] Brewer GJ, Boehler MD, Pearson RA, DeMaris AA, Ide AN, Wheeler BC (2008). Neuron network activity scales exponentially with synapse density. J Neural Eng.

[6] Buchser WJ, Slepak TI, Gutierrez-Arenas O, Bixby JL, Lemmon VP (2010). Kinase/phosphatase overexpression reveals pathways regulating hippocampal neuron morphology. Mol Syst Biol.

[7] Calafate S, Buist A, Miskiewicz K, Vijayan V, Daneels G, de Strooper B (2015). Synaptic contacts enhance cell-to-cell tau pathology propagation. Cell Rep.

[8] Callif BL, Maunze B, Krueger NL, Simpson MT, Blackmore MG (2017). The application of CRISPR technology to high content screening in primary neurons. Mol Cell Neurosci.

[9] Cooper DJ, Zunino G, Bixby JL, Lemmon VP (2017). Phenotypic screening with primary neurons to identify drug targets for regeneration and degeneration. Mol Cell Neurosci.

[10] Cornelissen F, Verstraelen P, Verbeke T, Pintelon I, Timmermans J-P, Nuydens R (2013). Quantitation of chronic and acute treatment effects on neuronal network activity using image and signal analysis: toward a high-content assay. J Biomol Screen.

[11] Coyle DE, Li J, Baccei M (2011). Regional differentiation of retinoic acid-induced human pluripotent embryonic carcinoma stem cell neurons. PLoS One.

[12] Detrez JR, Verstraelen P, Gebuis T, Verschuuren M, Kuijlaars J, Langlois X (2016). Image informatics strategies for deciphering neuronal network connectivity. Adv Anat Embryol Cell Biol.

[13] R Development Core Team R (2008) A language and environment for statistical computing. De Gruyter, Vienna. http://www.R-project.org. Accessed 22 May 2019.

[14] Di Z, Klop MJD, Rogkoti V-M, Le Dévédec SE, van de Water B, Verbeek FJ (2014). Ultra high content image analysis and phenotype profiling of 3D cultured micro-tissues. PLoS One.

[15] Dolmetsch R, Geschwind DH (2011). The human brain in a dish: the promise of iPSC-derived neurons. Cell.

[16] Falke E, Nissanov J, Mitchell TW, Bennett DA, Trojanowski JQ, Arnold SE (2003). Subicular dendritic arborization in Alzheimer's disease correlates with neurofibrillary tangle density. Am J Pathol.

[17] Floyd RA, Carney JM (1992). Free radical damage to protein and DNA: mechanisms involved and relevant observations on brain undergoing oxidative stress. Ann Neurol.

[18] Forster JI, Köglsberger S, Trefois C, Boyd O, Baumuratov AS, Buck L (2016). Characterization of differentiated SH-SY5Y as neuronal screening model reveals increased oxidative vulnerability. J Biomol Screen.

[19] Foster M, Sherrington CS (1897). A textbook of physiology.

[20] Francis JE, Mason D, Levy R (2017). Evaluation of quantum dot conjugated antibodies for immunofluorescent labelling of cellular targets. Beilstein J Nanotechnol.

[21] Frangi AF, Niessen WJ, Vincken KL, Viergever MA, Wells WM, Colchester A, Delp S (1998). Multiscale vessel enhancement filtering.

[22] Ghosh AS, Wang B, Pozniak CD, Chen M, Watts RJ, Lewcock JW (2011). DLK induces developmental neuronal degeneration via selective regulation of proapoptotic JNK activity. J Cell Biol.

[23] Gordon J, Amini S, White MK (2013). General overview of neuronal cell culture. Methods Mol Biol.

[24] Gräff J, Tsai L-H (2013). The potential of HDAC inhibitors as cognitive enhancers. Annu Rev Pharmacol Toxicol.

[25] Gunhanlar N, Shpak G, van der Kroeg M, Gouty-Colomer LA, Munshi ST, Lendemeijer B (2018). A simplified protocol for differentiation of electrophysiologically mature neuronal networks from human induced pluripotent stem cells. Mol Psychiatry.

[26] Gut G, Herrmann MD, Pelkmans L (2018). Multiplexed protein maps link subcellular organization to cellular states. Science.

[27] Haile Y, Fu W, Shi B, Westaway D, Baker G, Jhamandas J (2014). Characterization of the NT2-derived neuronal and astrocytic cell lines as alternative in vitro models for primary human neurons and astrocytes. J Neurosci Res.

[28] Harrill JA, Robinette BL, Mundy WR (2011). Use of high content image analysis to detect chemical-induced changes in synaptogenesis in vitro. Toxicol in Vitro.

[29] Hill EJ, Jiménez-González C, Tarczyluk M, Nagel DA, Coleman MD, Parri HR (2012). NT2 derived neuronal and astrocytic network signalling. PLoS One.

[30] A study of gdc-0134 to determine initial safety, tolerability, and pharmacokinetic parameters in participants with amyotrophic lateral sclerosis. 2019. https://www.clinicaltrials.gov/ct2/show/NCT02655614?term=GDC-0134. Accessed 22 May 2019.

[31] Hu M, Schurdak ME, Puttfarcken PS, Kouhen El R, Gopalakrishnan M, Li J (2007). High content screen microscopy analysis of a beta 1-42-induced neurite outgrowth reduction in rat primary cortical neurons: neuroprotective effects of alpha 7 neuronal nicotinic acetylcholine receptor ligands. Brain Res.

[32] Huang Y-WA, Zhou B, Wernig M, SUdhof TC (2017). ApoE2, ApoE3, and ApoE4 differentially stimulate APP transcription and Aβ secretion. Cell.

[33] Ichikawa M, Muramoto K, Kobayashi K, Kawahara M, Kuroda Y (1993). Formation and maturation of synapses in primary cultures of rat cerebral cortical-cells - an electron-microscopic study. Neurosci Res.

[34] Jaworski J, Sheng M (2006). The growing role of mTOR in neuronal development and plasticity. Mol Neurobiol.

[35] Kowalski JW, Gawel M, Pfeffer A, Barcikowska M (2001). The diagnostic value of EEG in Alzheimer disease - correlation with the severity of mental impairment. J Clin Neurophysiol.

[36] Kuijlaars J, Oyelami T, Diels A, Rohrbacher J, Versweyveld S, Meneghello G (2016). Sustained synchronized neuronal network activity in a human astrocyte co-culture system. Sci Rep.

[37] Laplante M, Sabatini DM (2012). mTOR signaling in growth control and disease. Cell.

[38] Le Pichon CE, Meilandt WJ, Dominguez S, Solanoy H, Lin H, Ngu H (2017). Loss of dual leucine zipper kinase signaling is protective in animal models of neurodegenerative disease. Sci Transl Med.

[39] Lin J-R, Fallahi-Sichani M, Sorger PK (2015). Highly multiplexed imaging of single cells using a high-throughput cyclic immunofluorescence method. Nat Commun.

[40] Lin Y-C, Koleske AJ (2010). Mechanisms of synapse and dendrite maintenance and their disruption in psychiatric and neurodegenerative disorders. Annu Rev Neurosci.

[41] Lipton SA (2006). Paradigm shift in neuroprotection by NMDA receptor blockade: Memantine and beyond. Nat Rev Drug Discov.

[42] Miller BR, Press C, Daniels RW, Sasaki Y, Milbrandt J, DiAntonio A (2009). A dual leucine kinase-dependent axon self-destruction program promotes Wallerian degeneration. Nat Neurosci.

[43] Nieland TJF, Logan DJ, Saulnier J, Lam D, Johnson C, Root DE (2014). High content image analysis identifies novel regulators of synaptogenesis in a high-throughput RNAi screen of primary neurons. PLoS One.

[44] O'Rourke NA, Weiler NC, Micheva KD, Smith SJ (2012). Deep molecular diversity of mammalian synapses: why it matters and how to measure it. Nat Rev Neurosci.

[45] Pani G, De Vos WH, Samari N, de Saint-Georges L, Baatout S, Van Oostveldt P (2014). MorphoNeuroNet: an automated method for dense neurite network analysis. Cytometry A.

[46] Patel S, Cohen F, Dean BJ, La Torre De K, Deshmukh G, Estrada AA (2015). Discovery of dual leucine zipper kinase (DLK, MAP3K12) inhibitors with activity in neurodegeneration models. J Med Chem.

[47] Patel S, Meilandt WJ, Erickson RI, Chen J, Deshmukh G, Estrada AA (2017). Selective inhibitors of dual leucine zipper kinase (DLK, MAP3K12) with activity in a model of Alzheimer's disease. J Med Chem.

[48] Patel TP, Man K, Firestein BL, Meaney DF (2015). Automated quantification of neuronal networks and single-cell calcium dynamics using calcium imaging. J Neurosci Methods.

[49] Pittenger C, Duman RS (2008). Stress, depression, and neuroplasticity: a convergence of mechanisms. Neuropsychopharmacology.

[50] Pozniak CD, Ghosh AS, Gogineni A, Hanson JE, Lee S-H, Larson JL (2013). Dual leucine zipper kinase is required for excitotoxicity-induced neuronal degeneration. J Exp Med.

[51] Reisberg B, Doody R, Stöffler A, Schmitt F, Ferris S, Möbius HJ (2003). Memantine in moderate-to-severe Alzheimer's disease. N Engl J Med.

[52] Sabatini DM, Erdjumentbromage H, Lui M, Tempst P, Snyder SH (1994). Raft1 - a mammalian protein that binds to Fkbp12 in a rapamycin-dependent fashion and is homologous to yeast tors. Cell.

[53] Sharma K, Choi S-Y, Zhang Y, Nieland TJF, Long S, Li M (2013). High-throughput genetic screen for Synaptogenic factors: identification of LRP6 as critical for excitatory synapse Development. Cell Rep.

[54] Siu Michael, Sengupta Ghosh Arundhati, Lewcock Joseph W. (2018). Dual Leucine Zipper Kinase Inhibitors for the Treatment of Neurodegeneration. Journal of Medicinal Chemistry.

[55] Spicer TP, Hubbs C, Vaissiere T, Collia D, Rojas C, Kilinc M (2018). Improved scalability of neuron-based phenotypic screening assays for therapeutic discovery in neuropsychiatric disorders. Mol Neuropsychiatry.

[56] Spilman P, Podlutskaya N, Hart MJ, Debnath J, Gorostiza O, Bredesen D (2010). Inhibition of mTOR by rapamycin abolishes cognitive deficits and reduces amyloid-beta levels in a mouse model of Alzheimer's disease. PLoS One.

[57] Stetter O, Battaglia D, Soriano J, Geisel T (2012). Model-free reconstruction of excitatory neuronal connectivity from calcium imaging signals. PLoS Comput Biol.

[58] Swiech L, Perycz M, Malik A, Jaworski J (2008). Role of mTOR in physiology and pathology of the nervous system. Biochim Biophys Acta.

[59] Taschenberger G, Toloe J, Tereshchenko J, Akerboom J, Wales P, Benz R (2013). beta-Synuclein aggregates and induces neurodegeneration in dopaminergic neurons. Ann Neurol.

[60] Terry RD, Masliah E, Salmon DP, Butters N, DeTeresa R, Hill R (1991). Physical basis of cognitive alterations in Alzheimer's disease: synapse loss is the major correlate of cognitive impairment. Ann Neurol.

[61] Valko M, Leibfritz D, Moncol J, Cronin MTD, Mazur M, Telser J (2007). Free radicals and antioxidants in normal physiological functions and human disease. Int J Biochem Cell Biol.

[62] Verstraelen P, Detrez JR, Verschuuren M, Kuijlaars J, Nuydens R, Timmermans J-P (2017). Dysregulation of microtubule stability impairs Morphofunctional connectivity in primary neuronal networks. Front Cell Neurosci.

[63] Verstraelen P, Pintelon I, Nuydens R, Cornelissen F, Meert T, Timmermans J-P (2014). Pharmacological characterization of cultivated neuronal networks: relevance to synaptogenesis and synaptic connectivity. Cell Mol Neurobiol.

[64] Verstraelen P, Van Dyck M, Verschuuren M, Kashikar ND, Nuydens R, Timmermans J-P, et al (2018) Image-based profiling of synaptic connectivity in primary neuronal cell culture. Front Neurosci 12 10.3389/fnins.2018.00389 PMC6028601 29997468

[65] Virdee JK, Saro G, Fouillet A, Findlay J, Ferreira F, Eversden S (2017). A high-throughput model for investigating neuronal function and synaptic transmission in cultured neuronal networks. Sci Rep.

[66] Watkins TA, Wang B, Huntwork-Rodriguez S, Yang J, Jiang Z, Eastham-Anderson J (2013). DLK initiates a transcriptional program that couples apoptotic and regenerative responses to axonal injury. Proc Natl Acad Sci U S A.

[67] Welsbie DS, Mitchell KL, Jaskula-Ranga V, Sluch VM, Yang Z, Kim J (2017). Enhanced functional genomic screening identifies novel mediators of dual leucine zipper kinase-dependent injury signaling in neurons. Neuron.

[68] Welsbie DS, Yang Z, Ge Y, Mitchell KL, Zhou X, Martin SE (2013). Functional genomic screening identifies dual leucine zipper kinase as a key mediator of retinal ganglion cell death. Proc Natl Acad Sci U S A.

[69] Yang S-S, Zhang R, Wang G, Zhang Y-F (2017). The development prospection of HDAC inhibitors as a potential therapeutic direction in Alzheimer’s disease. Transl Neurodegener.

[70] Yarza R, Vela S, Solas M, Ramirez MJ (2016). C-Jun N-terminal kinase (JNK) signaling as a therapeutic target for Alzheimer’s disease. Front Pharmacol.

[71] Yin C, Huang G-F, Sun X-C, Guo Z, Zhang JH (2017). DLK silencing attenuated neuron apoptosis through JIP3/MA2K7/JNK pathway in early brain injury after SAH in rats. Neurobiol Dis.

[72] Zhang L, Liu C, Wu J, Tao J-J, Sui X-L, Yao Z-G (2014). Tubastatin a/ACY-1215 improves cognition in Alzheimer's disease transgenic mice. J Alzheimers Dis.

[73] Zhou Q, Lam PY, Han D, Cadenas E (2009). Activation of c-Jun-N-terminal kinase and decline of mitochondrial pyruvate dehydrogenase activity during brain aging. FEBS Lett.

[74] Hsu Chia-Wen, Shou David, Huang Ruili, Khuc Thai, Dai Sheng, Zheng Wei, Klumpp-Thomas Carleen, Xia Menghang (2016). Identification of HDAC Inhibitors Using a Cell-Based HDAC I/II Assay. Journal of Biomolecular Screening.

[75] Parsons C. G., Panchenko V. A., Pinchenko V. O., Tsyndrenko A. Y., Krishtal O. A. (1996). Comparative Patch-clamp Studies with Freshly Dissociated Rat Hippocampal and Striatal Neurons on the NMDA Receptor Antagonistic Effects of Amantadine and Memantine. European Journal of Neuroscience.

[76] Ruan B., Pong K., Jow F., Bowlby M., Crozier R. A., Liu D., Liang S., Chen Y., Mercado M. L., Feng X., Bennett F., von Schack D., McDonald L., Zaleska M. M., Wood A., Reinhart P. H., Magolda R. L., Skotnicki J., Pangalos M. N., Koehn F. E., Carter G. T., Abou-Gharbia M., Graziani E. I. (2007). Binding of rapamycin analogs to calcium channels and FKBP52 contributes to their neuroprotective activities. Proceedings of the National Academy of Sciences.

[77] Zwick Vincent, Simões-Pires Claudia A., Nurisso Alessandra, Petit Charlotte, Dos Santos Passos Carolina, Randazzo Giuseppe Marco, Martinet Nadine, Bertrand Philippe, Cuendet Muriel (2016). Synthesis of a selective HDAC6 inhibitor active in neuroblasts. Bioorganic & Medicinal Chemistry Letters.

