# Supplementary material for: High-throughput microscopy exposes a pharmacological window in which dual leucine zipper kinase inhibition preserves neuronal network connectivity
Source: Acta Neuropathol Commun. 2019 Jun 4;7:6. doi: 10.1186/s40478-019-0741-3 (PMC6549294; doi:10.1186/s40478-019-0741-3)
Supplement: Supplementary file 1 — Table S1. All descriptors for the dendrite network, the synapse markers, the nuclei and functional (calcium) activity. Measurements are reported per field of view. (PDF 10993 kb) [file 40478_2019_741_MOESM1_ESM.pdf]

Additional file 1: **Table S1.** All descriptors for the dendrite network, the synapse markers, the nuclei and functional (calcium) activity. Measurements are reported per field of view.

Descriptor	Set	Explanation	Unit
ChannelDendrite_Contrast	Dendrites	Haralick texture descriptor	/
ChannelDendrite_Correlation	Dendrites	Haralick texture descriptor	/
ChannelDendrite_Homogeneity	Dendrites	Haralick texture descriptor	/
ChannelDendrite_Intensity	Dendrites	Haralick texture descriptor	/
ChannelDendrite_IntensityStddev	Dendrites	Haralick texture descriptor	/
ChannelDendrite_SumVariance	Dendrites	Haralick texture descriptor	/
Dendrite_Area	Dendrites	MAP2 positive area	μm^2
Dendrite_AreaPerNucleus	Dendrites	MAP2 positive area normalised to the number of neuronal nuclei	μm^2/nucleus
Dendrite_AreaSearch	Dendrites	Dilated MAP2 positive area with exclusion of neuronal nuclei	μm^2
Dendrite_Int	Dendrites	MAP2 intensity in the dendrite mask	AU
Dendrite_Int_sd	Dendrites	Standard deviation of the MAP2 intensity in the dendrite mask	/
Dendrite_IntSpot1	Dendrites	Synaptophysin intensity in the dendrite mask	AU
Dendrite_IntSpot2	Dendrites	PSD-95 intensity in the dendrite mask	AU
Dendrite_Length	Dendrites	Length of the skeletonised dendrite network	µm
Dendrite_LengthPerNucleus	Dendrites	Length of the skeletonised dendrite network normalised to the number of neuronal nuclei	µm/nucleus
Dendrite_Nodes	Dendrites	Number of branch points in the skeletonised dendrite network	#
Dendrite_Width_Max	Dendrites	Maximal width of the dendrites	µm
Dendrite_Width_Mean	Dendrites	Average width of the dendrites	µm
ChannelSpot1_Contrast	Synapses	Haralick texture descriptor	/
ChannelSpot1_Correlation	Synapses	Haralick texture descriptor	/
ChannelSpot1_Homogeneity	Synapses	Haralick texture descriptor	/
ChannelSpot1_Intensity	Synapses	Haralick texture descriptor	/
ChannelSpot1_IntensityStddev	Synapses	Haralick texture descriptor	/
ChannelSpot1_SumVariance	Synapses	Haralick texture descriptor	/
ChannelSpot2_Contrast	Synapses	Haralick texture descriptor	/
ChannelSpot2_Correlation	Synapses	Haralick texture descriptor	/
ChannelSpot2_Homogeneity	Synapses	Haralick texture descriptor	/
ChannelSpot2_Intensity	Synapses	Haralick texture descriptor	/
ChannelSpot2_IntensityStddev	Synapses	Haralick texture descriptor	/
ChannelSpot2_SumVariance	Synapses	Haralick texture descriptor	/
PearsonCorr	Synapses	Correlation of synaptophysin and PSD-95 signal within dendrite mask	/
SimilarityIndex	Synapses	Number of overlapping pre- and postsynaptic pixels devided by the sum of pre-and postsynaptic pixels	/
Spot1_Area	Synapses	Average area of presynaptic spots	μm^2
Spot1_Count	Synapses	Presynaptic count	#
Spot1_DensityArea	Synapses	Presynaptic count normalised to dendrite area	#/μm^2
Spot1_DensityLength	Synapses	Presynaptic count normalised to dendrite length	#/µm
Spot1_Int	Synapses	Average intensity of presynaptic spots	AU
Spot1_Overlap	Synapses	Average ratio of overlapping pixels with postsynaptic spots	/
Spot1_PercWithOverlap	Synapses	Percentage of presynaptic spots that overlap with postsynaptic spots	%
Spot2_Area	Synapses	Average area of postsynaptic spots	μm^2
Spot2_Count	Synapses	Postsynaptic count	#
Spot2_DensityArea	Synapses	Postsynaptic count normalised to dendrite area	#/μm^2
Spot2_DensityLength	Synapses	Postsynaptic count normalised to dendrite length	#/µm
Spot2_Int	Synapses	Average intensity of postsynaptic spots	AU
Spot2_Overlap	Synapses	Average ratio of overlapping pixels with presynaptic spots	/
Spot2_PercWithOverlap	Synapses	Percentage of postsynaptic spots that overlap with presynaptic spots	%
Synapses_Area	Synapses	Average area of synapses	μm^2
Synapses_Count	Synapses	Synaptic count	#
Synapses_Density	Synapses	Synaptic count normalised to dendrite area	#/μm^2
Synapses_IntRatio	Synapses	Intensity of synaptophysin over the intensity of PSD-95 in synapses	/
Spot1_IntBg	Synapses	Background intensity in dendrite mask of synaptophysin	AU
Spot2_IntBg	Synapses	Background intensity in dendrite mask of PSD-95	AU
Spot1_SBR	Synapses	Signal to background ratio of synaptophysin	/
Spot2_SBR	Synapses	Signal to background ratio of PSD-95	/
ChannelNuclei_Contrast	Nuclei	Haralick texture descriptor	/
ChannelNuclei_Correlation	Nuclei	Haralick texture descriptor	/
ChannelNuclei_Homogeneity	Nuclei	Haralick texture descriptor	/
ChannelNuclei_Intensity	Nuclei	Haralick texture descriptor	/
ChannelNuclei_IntensityStddev	Nuclei	Haralick texture descriptor	/
ChannelNuclei_SumVariance	Nuclei	Haralick texture descriptor	/
Nuclei_Area	Nuclei	Average area of nuclei	μm^2
Nuclei_Count	Nuclei	Nuclear count	#
Nuclei_Int	Nuclei	Average intensity of nuclei	AU
Nuclei_Int_relDev	Nuclei	Average standard deviation of intensities within nuclei	/
Nuclei_IntNeu	Nuclei	Average intensity of MAP2 within nuclear mask	AU
Nuclei_IntSpot1	Nuclei	Average intensity of synaptophysin within nuclear mask	AU
Nuclei_IntSpot2	Nuclei	Average intensity of PSD95 within nuclear mask	AU

Nuclei_Neuronal_Area	Nuclei	Average area of neuronal nuclei	μm^2
Nuclei_Neuronal_Count	Nuclei	Neuronal nuclear count	#
Nuclei_Neuronal_Int	Nuclei	Average intensity of neuronal nuclei	AU
Nuclei_Neuronal_Int_relDev	Nuclei	Average standard deviation of intensities within neuronal nuclei	/
Nuclei_Neuronal_IntNeu	Nuclei	Average intensity of MAP2 within neuronal nuclear mask	AU
Nuclei_Neuronal_IntSpot1	Nuclei	Average intensity of synaptophysin within neuronal nuclear mask	AU
Nuclei_Neuronal_IntSpot2	Nuclei	Average intensity of PSD95 within neuronal nuclear mask	AU
Nuclei_Neuronal_Perimeter	Nuclei	Average perimeter of neuronal nuclei	µm
Nuclei_Neuronal_Ratio	Nuclei	Ratio of neuronal nuclei	/
Nuclei_Neuronal_Roundness	Nuclei	Average roundness of neuronal nuclei	/
Nuclei_Neuronal_SERSpot	Nuclei	Average spot-like texture of neuronal nuclei	/
Nuclei_NonNeuronal_Area	Nuclei	Average area of non-neuronal nuclei	μm^2
Nuclei_NonNeuronal_Count	Nuclei	Non-neuronal nuclear count	#
Nuclei_NonNeuronal_Int	Nuclei	Average intensity of non-neuronal nuclei	AU
Nuclei_NonNeuronal_Int_relDev	Nuclei	Average standard deviation of intensities within non-neuronal nuclei	/
Nuclei_NonNeuronal_IntNeu	Nuclei	Average intensity of MAP2 within non-neuronal nuclear mask	AU
Nuclei_NonNeuronal_IntSpot1	Nuclei	Average intensity of synaptophysin within non-neuronal nuclear mask	AU
Nuclei_NonNeuronal_IntSpot2	Nuclei	Average intensity of PSD95 within non-neuronal nuclear mask	AU
Nuclei_NonNeuronal_Perimeter	Nuclei	Average perimeter of non-neuronal nuclei	µm
Nuclei_NonNeuronal_Ratio	Nuclei	Ratio of non-neuronal nuclei	/
Nuclei_NonNeuronal_Roundness	Nuclei	Average roundness of non-neuronal nuclei	/
Nuclei_NonNeuronal_SERSpot	Nuclei	Average spot-like texture of non-neuronal nuclei	/
Nuclei_Perimeter	Nuclei	Average perimeter of nuclei	/
Nuclei_Roundness	Nuclei	Average roundness of nuclei	/
Nuclei_SERSpot	Nuclei	Average spot-like texture of nuclei	/
CA_cMeanAvBurstAmpl	Functional	Average burst amplitude	AU
CA_cMeanAvBurstInterval	Functional	Average burst interval	s
CA_cMeanAvDecayTime	Functional	Average burst decay time	s
CA_cMeanBurstFreq	Functional	Average burst frequency	#/min
CA_meanCorr	Functional	Correlation of bursts	/
CA_pctActiveneurons	Functional	Percentage of active neurons	%
CA_sMeanBurstInterval	Functional	Average burst amplitude	s
CA_sSyncAvBurstAmpl	Functional	Average burst amplitude of synchronous bursts	AU
CA_sSyncBurstFreq	Functional	Average burst frequency of synchronous bursts	s
CA_sSyncMeanDecayTime	Functional	Average burst decay time of synchronous bursts	#/min
