# Supplementary material for: High-throughput microscopy exposes a pharmacological window in which dual leucine zipper kinase inhibition preserves neuronal network connectivity
Source: Acta Neuropathol Commun. 2019 Jun 4;7:6. doi: 10.1186/s40478-019-0741-3 (PMC6549294; doi:10.1186/s40478-019-0741-3)
Supplement: Supplementary file 2 — Figure S1. Opera Phenix system chromatic aberration and overlap criteria of pre- and postsynaptic markers. (a) Fluorescent beads of 0.1 and 0.5 μm diameter were recorded with a 40x water immersion objective (NA = 1.1) at an image resolution of 0.149 μm/pixel. Dashed white lines show the axis at which the intensities of the 488 nm (typically used for PSD95) and 561 nm (typically used for Synaptophysin) excitation channel were measured. Quantifications in XY and XZ show that the shift between both channels lies below 1 pixel for both bead types. (n = 25 beads); (b) An interpolated shift of 0.6 pixel (~ shift calculated for 0.1 μm beads in panel (a)) or a one-pixel shift of the PSD95 channel to correct for the chromatic aberration does not alter the synapse density calculation in images of cortical cultures, fixed at 3/7/10/14/18 DIV (ntech = 6); (c) Different criteria for synapse detection (1, 2 or 3 pixels overlap between pre- and postsynaptic spots) alter the absolute synapse numbers slightly, but do not affect the relative change across DIVs (ntech = 6). (PDF 10993 kb) [file 40478_2019_741_MOESM2_ESM.pdf]

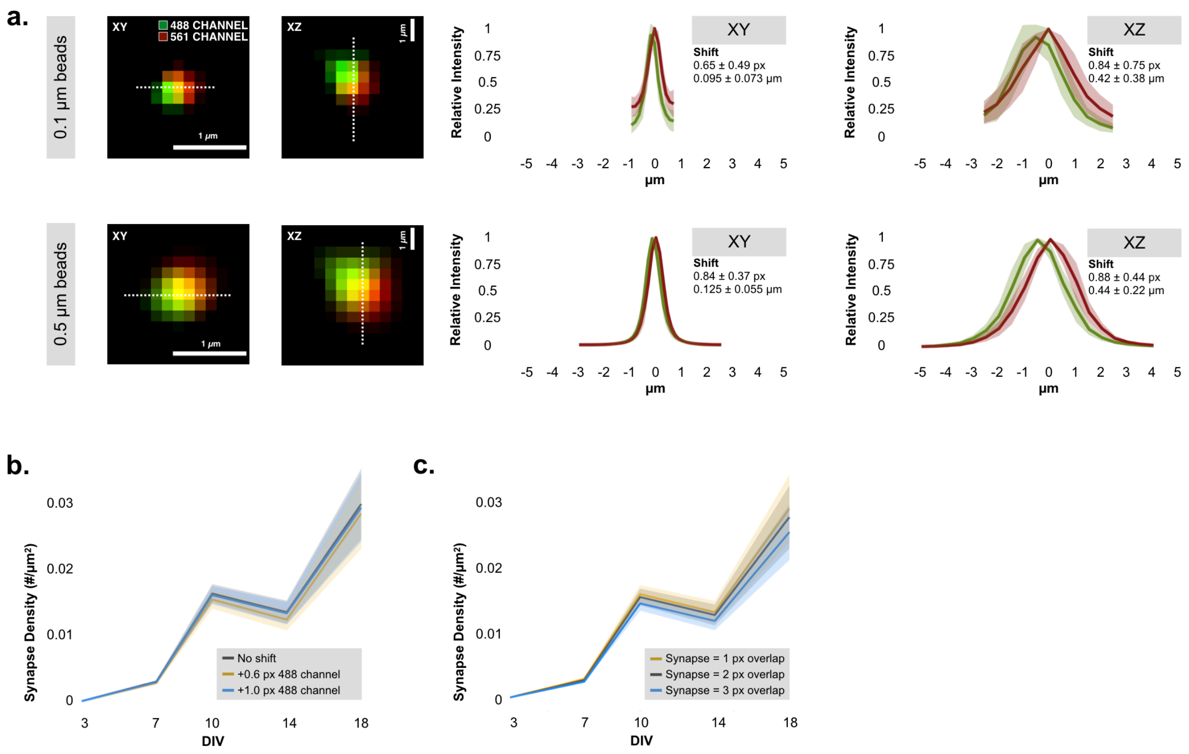

Additional file 2: **Figure S1**. Opera Phenix system chromatic aberration and overlap criteria of pre- and postsynaptic markers. **(a)** Fluorescent beads of 0.1 and 0.5 µm diameter were recorded with a 40x water immersion objective (NA = 1.1) at an image resolution of 0.149 µm/pixel. Dashed white lines show the axis at which the intensities of the 488 nm (typically used for PSD95) and 561 nm (typically used for Synaptophysin) excitation channel were measured. Quantifications in XY and XZ show that the shift between both channels lies below 1 pixel for both bead types. (n = 25 beads); **(b)** An interpolated shift of 0.6 pixel (~ shift calculated for 0.1 µm beads in panel (a)) or a one-pixel shift of the PSD95 channel to correct for the chromatic aberration does not alter the synapse density calculation in images of cortical cultures, fixed at 3/7/10/14/18 DIV (n_{tech} = 6); **(c)** Different criteria for synapse detection (1, 2 or 3 pixels overlap between pre- and postsynaptic spots) alter the absolute synapse numbers slightly, but do not significantly affect the change across DIVs (n_{tech} = 6).
