# Supplementary material for: High-throughput microscopy exposes a pharmacological window in which dual leucine zipper kinase inhibition preserves neuronal network connectivity
Source: Acta Neuropathol Commun. 2019 Jun 4;7:6. doi: 10.1186/s40478-019-0741-3 (PMC6549294; doi:10.1186/s40478-019-0741-3)
Supplement: Supplementary file 3 — Figure S2. Descriptor selection for the connectivity score. A subset of descriptors was selected based on the correlation with the DIV and inter-correlation between descriptors. First, the descriptors were ranked according to their correlation with the culture age. This rank was used to subsequently add descriptors to the final subset, making sure that the inter-correlation within descriptors of the subset did not exceed 0.75. Intensity descriptors were excluded from the descriptor set, because they were too sensitive to outliers. (PDF 10993 kb) [file 40478_2019_741_MOESM3_ESM.pdf]

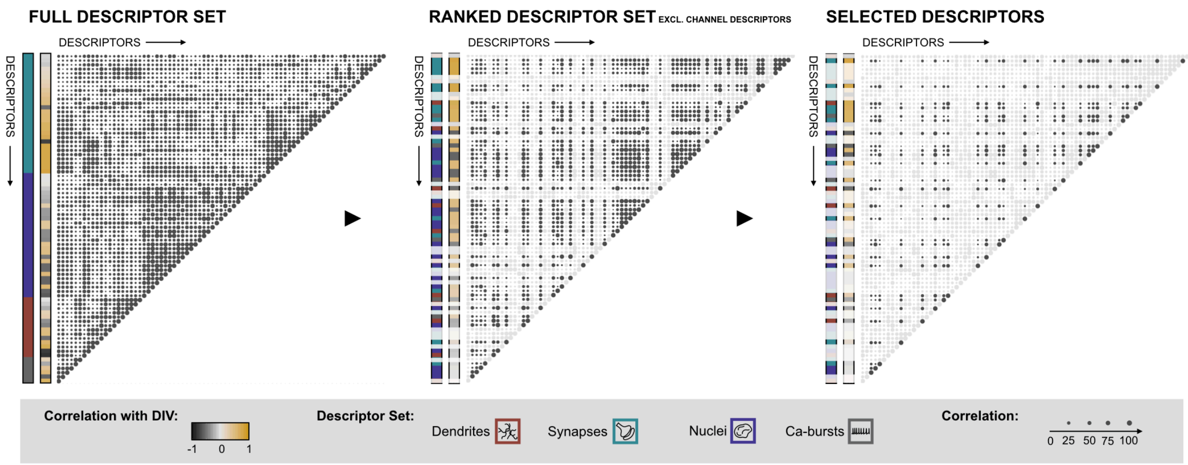

Additional file 3: **Figure S2**. Descriptor selection for the connectivity score. A subset of descriptors was selected based on the correlation with the DIV and inter-correlation between descriptors. First, the descriptors were ranked according to their correlation with the culture age. This rank was used to subsequently add descriptors to the final subset, making sure that the inter-correlation within descriptors of the subset did not exceed 0.75. Intensity descriptors were excluded from the descriptor set, because they were too sensitive to outliers.
