# Supplementary material for: High-throughput microscopy exposes a pharmacological window in which dual leucine zipper kinase inhibition preserves neuronal network connectivity
Source: Acta Neuropathol Commun. 2019 Jun 4;7:6. doi: 10.1186/s40478-019-0741-3 (PMC6549294; doi:10.1186/s40478-019-0741-3)
Supplement: Supplementary file 4 — Figure S3. Culture age correlates with morphological changes. Individual channels and composite of representative images of cortical cultures after fixation and immunocytochemistry at 6, 12, 18, 24, 30, 36, 42 and 48 DIV. (PDF 10993 kb) [file 40478_2019_741_MOESM4_ESM.pdf]

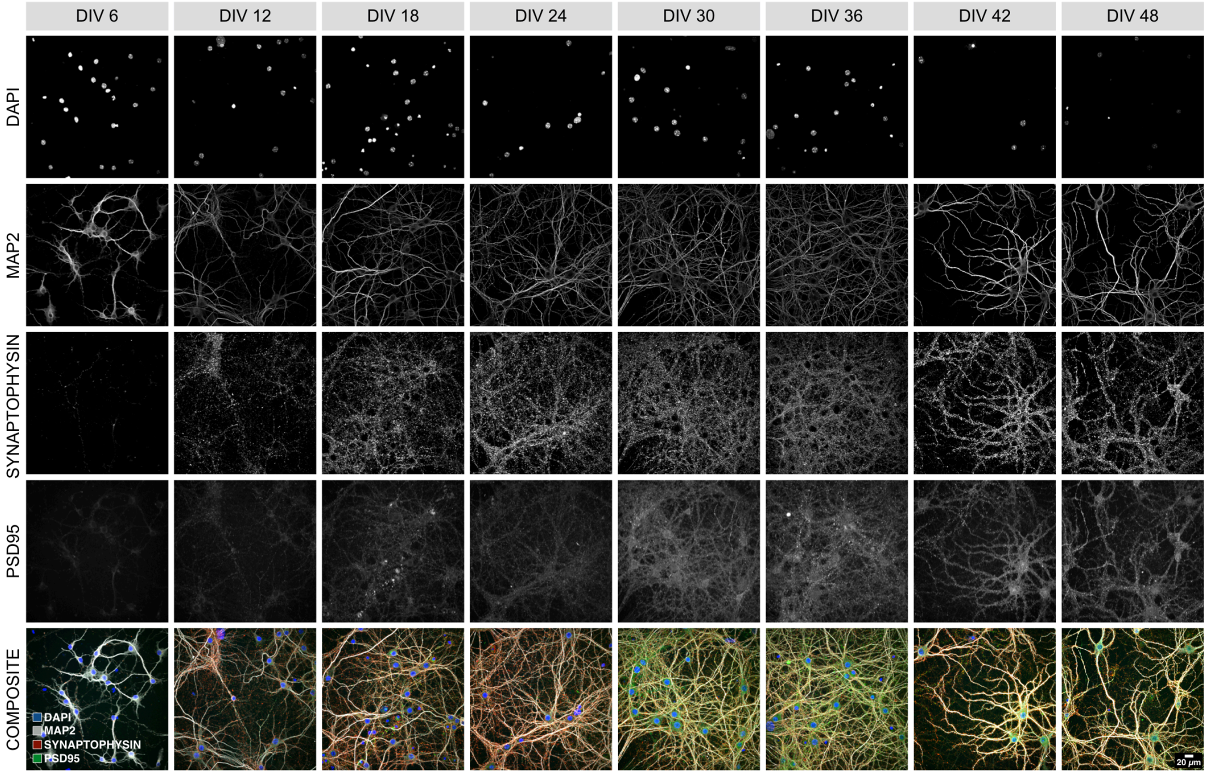

Additional file 4: **Figure S3.** Culture age correlates with morphological changes. Individual channels and composite of representative images of cortical cultures after fixation and immunocytochemistry at 6, 12, 18, 24, 30, 36, 42 and 48 DIV.
