# Supplementary material for: High-throughput microscopy exposes a pharmacological window in which dual leucine zipper kinase inhibition preserves neuronal network connectivity
Source: Acta Neuropathol Commun. 2019 Jun 4;7:6. doi: 10.1186/s40478-019-0741-3 (PMC6549294; doi:10.1186/s40478-019-0741-3)
Supplement: Supplementary file 5 — Figure S4. Differences between hippocampal and cortical cultures. (a) Dendrite width and postsynaptic intensity are higher in hippocampal cultures. The ratio of neuronal nuclei is higher in cortical cultures compared to hippocampal cultures that have more non-neuronal nuclei. This is reflected in the average texture and area of the nuclei, since neuronal nuclei are smaller and have a more spot-like phenotype (stronger texture) than non-neuronal nuclei (nbio = 3 x ntech = 6); (b) Representative images of both neuronal (red) and non-neuronal (yellow) nuclei in cortical and hippocampal cultures. (PDF 10993 kb) [file 40478_2019_741_MOESM5_ESM.pdf]

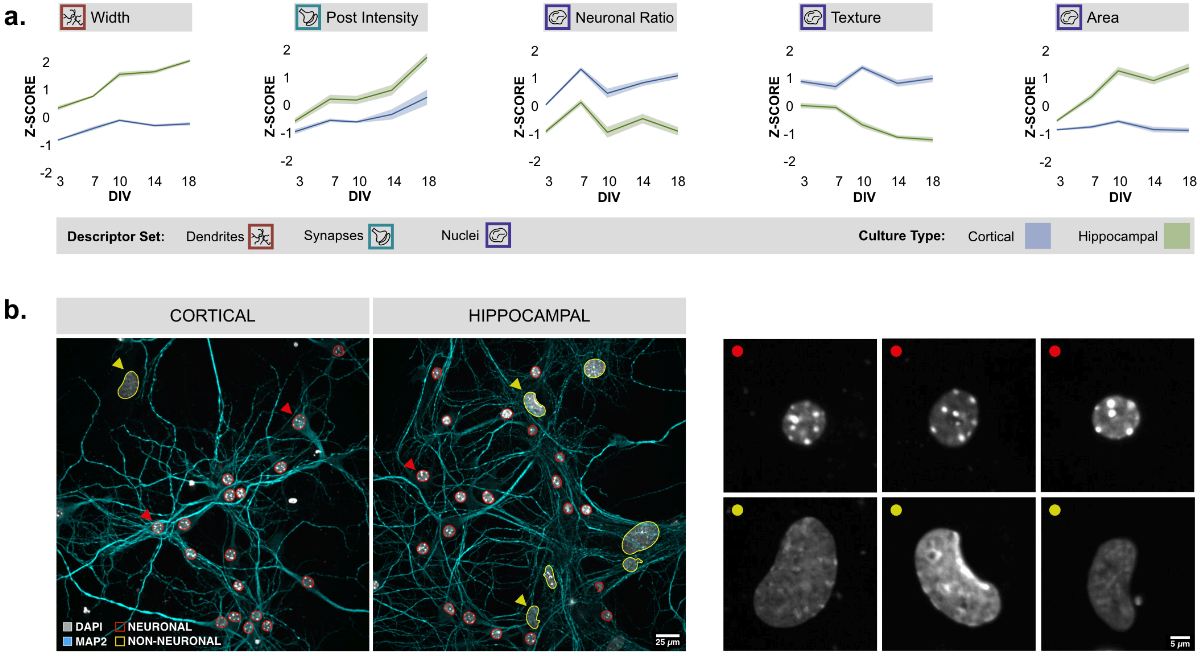

Additional file 5: **Figure S4.** Differences between hippocampal and cortical cultures. **(a)** Dendrite width and postsynaptic intensity are higher in hippocampal cultures. The ratio of neuronal nuclei is higher in cortical cultures compared to hippocampal cultures that have more non-neuronal nuclei. This is reflected in the average texture and area of the nuclei, since neuronal nuclei are smaller and have a more spot-like phenotype (stronger texture) than non-neuronal nuclei ($n_{bio} = 3$ x $n_{tech} = 6$); **(b)** Representative images of both neuronal (red) and non-neuronal (yellow) nuclei in cortical and hippocampal cultures.
