# Supplementary material for: High-throughput microscopy exposes a pharmacological window in which dual leucine zipper kinase inhibition preserves neuronal network connectivity
Source: Acta Neuropathol Commun. 2019 Jun 4;7:6. doi: 10.1186/s40478-019-0741-3 (PMC6549294; doi:10.1186/s40478-019-0741-3)
Supplement: Supplementary file 6 — Figure S5. Despite inter-replicate variability, cultures can be clustered and accurately classified. (a) Representative images of both hippocampal and cortical cultures at different DIV for 3 different replicates; (b) PCA based on different descriptor sets for individual replicates. PCA based on the integrated descriptor set resulted in the best clustering (nbio = 3 x ntech = 6) (Ellipses show the 67% confidence interval); (c) Confusion matrices of classification results using a random forest. The misclassification rate (MCR) was lowest using the integrated morphological descriptor set (nbio = 3 x ntech = 6); (d) Confusion matrices of classification results using a linear discriminant analysis. The MCR, compared to RFC, was lower for morphological data, but higher for functional data (nbio = 3 x ntech = 6). (PDF 10993 kb) [file 40478_2019_741_MOESM6_ESM.pdf]

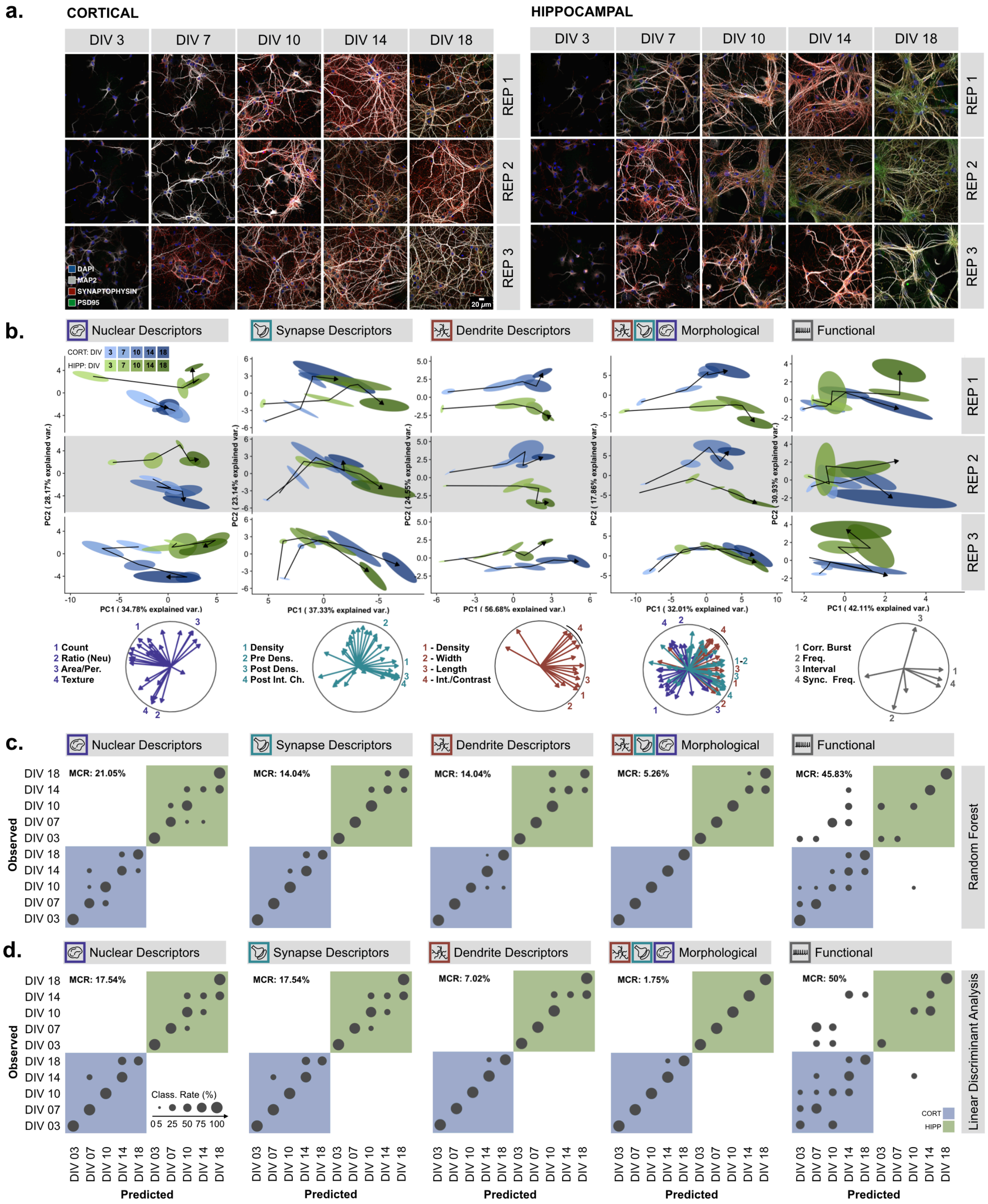

Additional file 6: **Figure S5**. Despite inter-replicate variability, cultures can be clustered and accurately classified. **(a)** Representative images of both hippocampal and cortical cultures at different DIV for 3 different replicates; **(b)** PCA based on different descriptor sets for individual replicates. PCA based on the integrated descriptor set resulted in the best clustering ($n_{bio} = 3$ x $n_{tech} = 6$) (Ellipses show the 67% confidence interval); **(c)** Confusion matrices of classification

results using a random forest. The misclassification rate (MCR) was lowest using the integrated morphological descriptor set ($n_{bio} = 3 \times n_{tech} = 6$); **(d)** Confusion matrices of classification results using a linear discriminant analysis. The MCR, compared to RFC, was lower for morphological data, but higher for functional data ($n_{bio} = 3 \times n_{tech} = 6$).
