# Supplementary material for: High-throughput microscopy exposes a pharmacological window in which dual leucine zipper kinase inhibition preserves neuronal network connectivity
Source: Acta Neuropathol Commun. 2019 Jun 4;7:6. doi: 10.1186/s40478-019-0741-3 (PMC6549294; doi:10.1186/s40478-019-0741-3)
Supplement: Supplementary file 7 — Figure S6. Functional descriptors entail unique information about neuronal connectivity. MK801 treatment (yellow) impaired the functional activity significantly at 18 DIV, that was not reflected in the morphological data (Morph.: nbio = 3 x ntech = 5 - Func.: nbio = 3 x ntech = 6). Significant differences between control and treated cultures are indicated (p < 0.05, pairwise Wilcoxon test with Bonferroni correction). (PDF 10993 kb) [file 40478_2019_741_MOESM7_ESM.pdf]

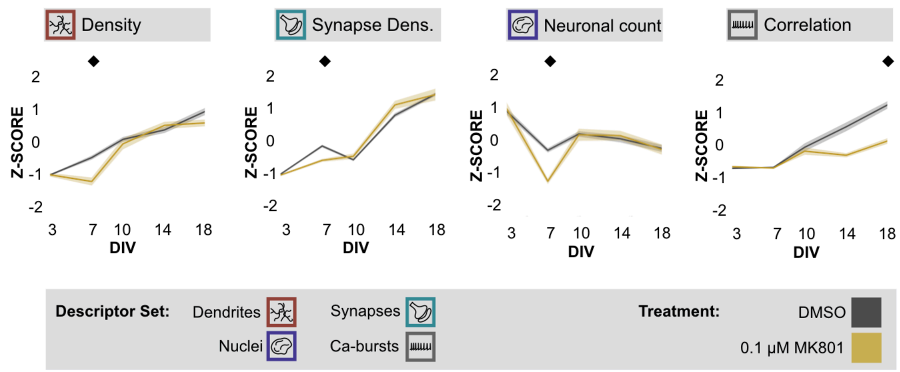

Additional file 7: **Figure S6**. Functional descriptors entail unique information about neuronal connectivity. MK801 treatment (yellow) impaired the functional activity significantly at 18 DIV, that was not reflected in the morphological data (Morph.: $n_{bio} = 3 \times n_{tech} = 5$ - Func.: $n_{bio} = 3 \times n_{tech} = 6$). Significant differences between control and treated cultures are indicated ($p<0.05$, pairwise Wilcoxon test with Bonferroni correction).
