# Supplementary material for: High-throughput microscopy exposes a pharmacological window in which dual leucine zipper kinase inhibition preserves neuronal network connectivity
Source: Acta Neuropathol Commun. 2019 Jun 4;7:6. doi: 10.1186/s40478-019-0741-3 (PMC6549294; doi:10.1186/s40478-019-0741-3)
Supplement: Supplementary file 8 — Figure S7. Nuclear descriptors entail unique information. AraC treatment (yellow) had a major negative impact on nuclear descriptors during the whole time range, while other descriptors showed only transient effects (e.g., dendrite density) or negative effects on later time points (e.g., correlation of the calcium bursts) (Morph.: nbio = 3 x ntech = 6 - Func.: nbio = 3 x ntech = 6). Significant differences between control and treated cultures are indicated (p < 0.05, pairwise Wilcoxon test with Bonferroni correction). (PDF 10993 kb) [file 40478_2019_741_MOESM8_ESM.pdf]

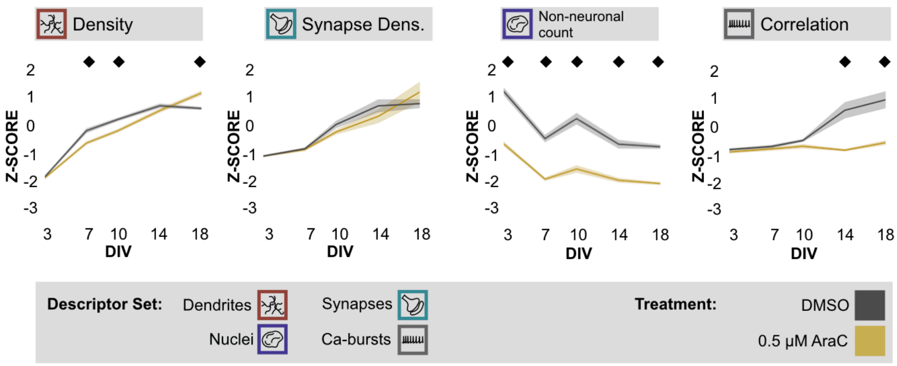

Additional file 8: **Figure S7**. Nuclear descriptors entail unique information. AraC treatment (yellow) had a major negative impact on nuclear descriptors during the whole time range, while other descriptors showed only transient effects (*e.g.,* dendrite density) or negative effects on later time points (*e.g.,* correlation of the calcium bursts) (Morph.: $n_{bio} = 3$ x $n_{tech} = 6$ - Func.: $n_{bio} = 3$ x $n_{tech} = 6$). Significant differences between control and treated cultures are indicated ($p<0.05$, pairwise Wilcoxon test with Bonferroni correction).
