# Supplementary material for: High-throughput microscopy exposes a pharmacological window in which dual leucine zipper kinase inhibition preserves neuronal network connectivity
Source: Acta Neuropathol Commun. 2019 Jun 4;7:6. doi: 10.1186/s40478-019-0741-3 (PMC6549294; doi:10.1186/s40478-019-0741-3)
Supplement: Supplementary file 9 — Figure S8. Connectivity scores are sensitive to changes in dendrite, synapse, nuclear and functional descriptors. (a) Connectivity scores of MK801-treated cultures showed greater differences with DMSO-treated cultures at later time points when based on the integrated dataset when compared to the scores only based on morphological data. (Morph.: nbio = 3 x ntech = 5 - Func.: nbio = 3 x ntech = 6); (b) Connectivity scores of AraC treated cultures revealed greater connectivity impairments in comparison with DMSO treated cultures when including nuclear descriptors (Morph.: nbio = 3 x ntech = 6 - Func.: nbio = 3 x ntech = 6). (PDF 10993 kb) [file 40478_2019_741_MOESM9_ESM.pdf]

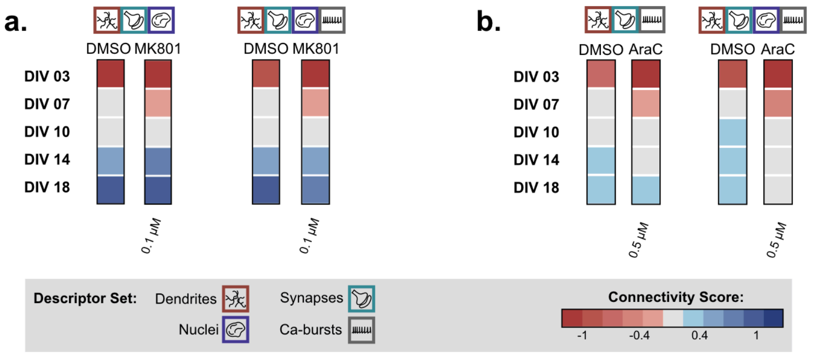

Additional file 9: **Figure S8**. Connectivity scores are sensitive to changes in dendrite, synapse, nuclear and functional descriptors. **(a)** Connectivity scores of MK801-treated cultures showed greater differences with DMSO-treated cultures at later time points when based on the integrated dataset when compared to the scores only based on morphological data. (Morph.: $n_{bio} = 3 \times n_{tech} = 5$ - Func.: $n_{bio} = 3 \times n_{tech} = 6$); **(b)** Connectivity scores of AraC treated cultures revealed greater connectivity impairments in comparison with DMSO treated cultures when including nuclear descriptors (Morph.: $n_{bio} = 3 \times n_{tech} = 6$ - Func.: $n_{bio} = 3 \times n_{tech} = 6$).
