# Supplementary material for: High-throughput microscopy exposes a pharmacological window in which dual leucine zipper kinase inhibition preserves neuronal network connectivity
Source: Acta Neuropathol Commun. 2019 Jun 4;7:6. doi: 10.1186/s40478-019-0741-3 (PMC6549294; doi:10.1186/s40478-019-0741-3)
Supplement: Supplementary file 10 — Figure S9. Classification of morphological data confirms findings based on connectivity score. A RFC that was trained on pooled DMSO treated cultures revealed a negative impact of rapamycin on neuronal connectivity as can be seen from the cultures that were misclassified and were assigned a culture age that was lower than the actual culture age (red). Treatment with 0.01 μM and 0.1 μM of GNE3511 could however improve the neuronal connectivity (green) (nbio = 3 x ntech = 5 except for GNE3511: nbio = 2 x ntech = 6). (PDF 10993 kb) [file 40478_2019_741_MOESM10_ESM.pdf]

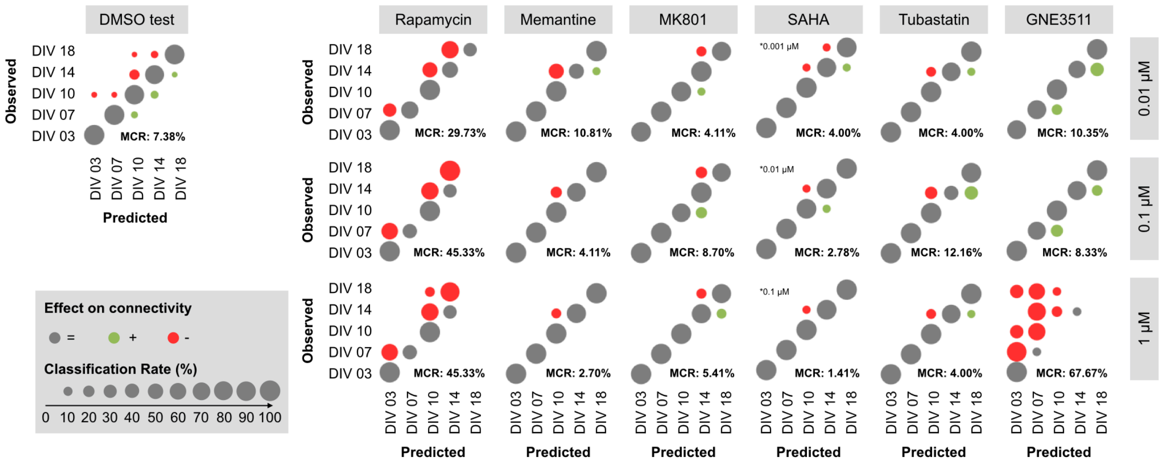

Additional file 10: **Figure S9**. Classification of morphological data confirms findings based on connectivity score. A RFC that was trained on pooled DMSO treated cultures revealed a negative impact of rapamycin on neuronal connectivity as can be seen from the cultures that were misclassified and were assigned a culture age that was lower than the actual culture age (red). Treatment with 0.01µM and 0.1 µM of GNE3511 could however improve the neuronal connectivity (green) (n_{bio}=3 x n_{tech}=5 except for GNE3511: n_{bio}=2 x n_{tech}=6).
