# Supplementary material for: High-throughput microscopy exposes a pharmacological window in which dual leucine zipper kinase inhibition preserves neuronal network connectivity
Source: Acta Neuropathol Commun. 2019 Jun 4;7:6. doi: 10.1186/s40478-019-0741-3 (PMC6549294; doi:10.1186/s40478-019-0741-3)
Supplement: Supplementary file 11 — Figure S10. Extended culture age reduces neuronal connectivity. Connectivity scores based on z-scores from cortical cultures grown for an extended period of time. Neuronal connectivity increased during the first two weeks, after which it stagnated up to five and a half weeks. From DIV 38 onwards age-related loss of neuronal connectivity was detected (Morph.: nbio = 1 x ntech = 6 - Func.: nbio = 1 x ntech = 9). (PDF 10993 kb) [file 40478_2019_741_MOESM11_ESM.pdf]

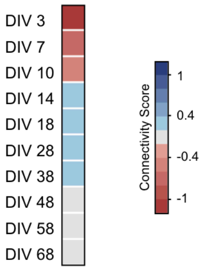

Additional file 11: **Figure S10**. Extended culture age reduces neuronal connectivity**.** Connectivity scores based on z-scores from cortical cultures grown for an extended period of time. Neuronal connectivity increased during the first two weeks, after which it stagnated up to five and a half weeks. From DIV 38 onwards age-related loss of neuronal connectivity was detected (Morph.: $n_{bio} = 1 \times n_{tech} = 6$ - Func.: $n_{bio} = 1 \times n_{tech} = 9$).
