# Supplementary material for: High-throughput microscopy exposes a pharmacological window in which dual leucine zipper kinase inhibition preserves neuronal network connectivity
Source: Acta Neuropathol Commun. 2019 Jun 4;7:6. doi: 10.1186/s40478-019-0741-3 (PMC6549294; doi:10.1186/s40478-019-0741-3)
Supplement: Supplementary file 12 — Figure S11. Impaired neuronal connectivity in suboptimal conditions. (a) Connectivity scores indicated that antioxidant deprivation (-AO) in primary cultures had a negative impact on neuronal network connectivity form 7 DIV onwards (Morph.: nbio = 2 x ntech = 6 - Func.: nbio = 2 x ntech = 9); (b) A RFC that was trained on morphological data of pooled DMSO treated cultures confirmed the negative impact of antioxidant deprivation (red) (nbio = 2 x ntech = 6); (c) Cultures overexpressing hTau.P301L, showed a decreasing neuronal connectivity from 10 DIV (Morph.: nbio = 2 x ntech = 12 - Func.: nbio = 2 x ntech = 9); (d) Classification results based on morphological data confirmed the negative effect of hTau.P301L overexpression on neuronal connectivity (red) (nbio = 2 x ntech = 12). (PDF 10993 kb) [file 40478_2019_741_MOESM12_ESM.pdf]

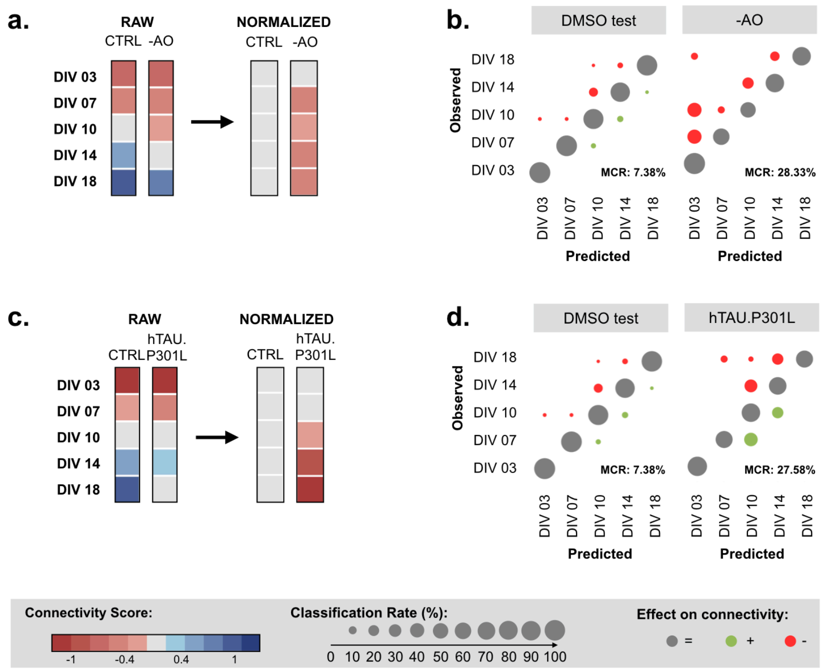

Additional file 12: **Figure S11.** Impaired neuronal connectivity in suboptimal conditions. **(a)** Connectivity scores indicated that antioxidant deprivation (-AO) in primary cultures had a negative impact on neuronal network connectivity form 7 DIV onwards (Morph.: $n_{bio} = 2 \times n_{tech} = 6$ - Func.: $n_{bio} = 2 \times n_{tech} = 9$); **(b)** A RFC that was trained on morphological data of pooled DMSO treated cultures confirmed the negative impact of antioxidant deprivation (red) ($n_{bio} = 2 \times n_{tech} = 6$); **(c)** Cultures overexpressing hTau.P301L, showed a decreasing neuronal connectivity from 10 DIV (Morph.: $n_{bio} = 2 \times n_{tech} = 12$ - Func.: $n_{bio} = 2 \times n_{tech} = 9$); **(d)** Classification results based on morphological data confirmed the negative effect of hTau.P301L overexpression on neuronal connectivity (red) ($n_{bio} = 2 \times n_{tech} = 12$).
