# Supplementary material for: High-throughput microscopy exposes a pharmacological window in which dual leucine zipper kinase inhibition preserves neuronal network connectivity
Source: Acta Neuropathol Commun. 2019 Jun 4;7:6. doi: 10.1186/s40478-019-0741-3 (PMC6549294; doi:10.1186/s40478-019-0741-3)
Supplement: Supplementary file 13 — Figure S12. Western blot analyses of (phosphorylated) Jun and AT8. (a) Western blot showed an increase in total c-Jun and phosphorylated c-Jun (Ser 63) in cultures overexpressing hTAU.P301L. Treatment with GNE8505 reduced c-Jun and phosphorylated c-Jun in control, antioxidant deprived (-AO) and hTau.P301L cultures (excl. Phosphorylated c-Jun Ser 63 in control and -AO cultures) (nbio = 1 x ntech = 1); (b) Western blot showed an increase in hyperphosphorylated (AT8) tau in cultures overexpressing hTau.P301L (nbio = 1 x ntech = 1). (PDF 10993 kb) [file 40478_2019_741_MOESM13_ESM.pdf]

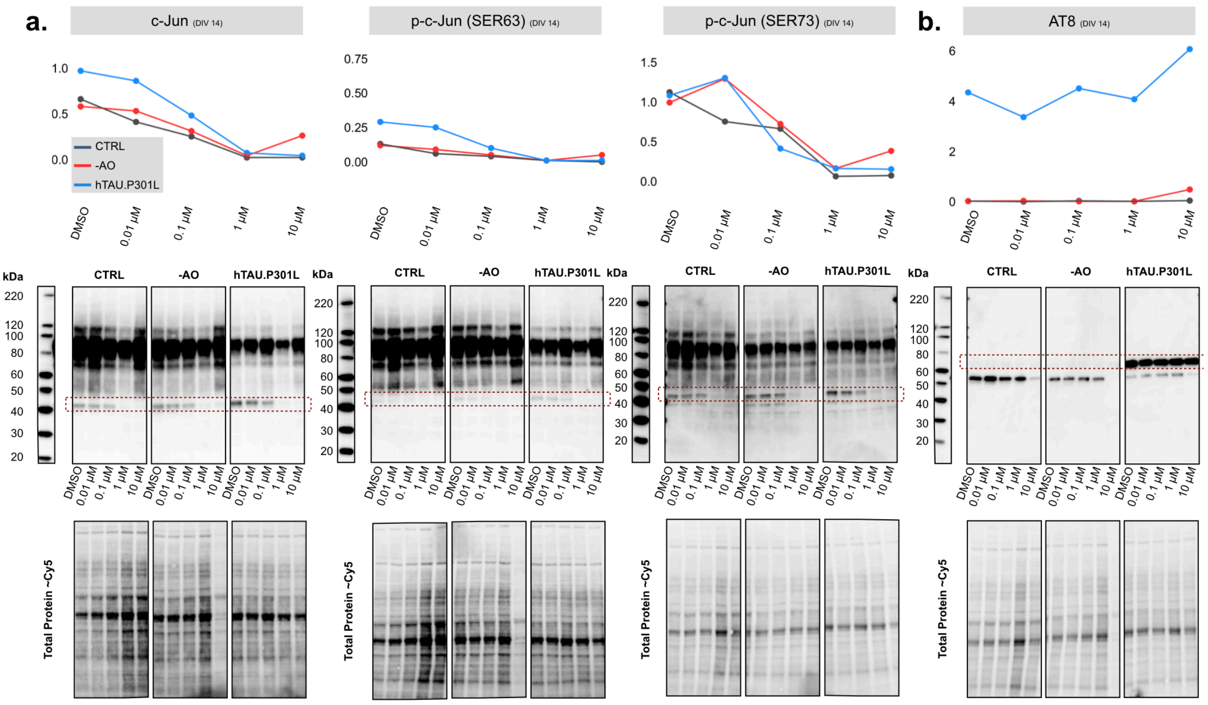

Additional file 13: **Figure S12.** Western blot analyses of (phosphorylated) Jun and AT8. **(a)** Western blot showed an increase in total c-Jun and phosphorylated c-Jun (Ser 63) in cultures overexpressing hTau.P301L. Treatment with GNE8505 reduced c-Jun and phosphorylated c-Jun in control, antioxidant deprived (-AO) and hTau.P301L cultures (excl. phosphorylated c-Jun Ser 63 in control and -AO cultures) ($n_{bio} = 1$ x $n_{tech} = 1$); **(b)** Western blot showed an increase in hyperphosphorylated (AT8) tau in cultures overexpressing hTau.P301L ($n_{bio} = 1$ x $n_{tech} = 1$).
